# Genetic Determinants Influencing Human Serum Metabolome among African Americans

**DOI:** 10.1371/journal.pgen.1004212

**Published:** 2014-03-13

**Authors:** Bing Yu, Yan Zheng, Danny Alexander, Alanna C. Morrison, Josef Coresh, Eric Boerwinkle

**Affiliations:** 1 Human Genetics Center, University of Texas Health Science Center at Houston, Houston, Texas, United States of America; 2 Metabolon, Inc., Durham, North Carolina, United States of America; 3 Department of Epidemiology, Johns Hopkins University, Baltimore, Maryland, United States of America; 4 Human Genome Sequencing Center, Baylor College of Medicine, Houston, Texas, United States of America; Georgia Institute of Technology, United States of America

## Abstract

Phenotypes proximal to gene action generally reflect larger genetic effect sizes than those that are distant. The human metabolome, a result of multiple cellular and biological processes, are functional intermediate phenotypes proximal to gene action. Here, we present a genome-wide association study of 308 untargeted metabolite levels among African Americans from the Atherosclerosis Risk in Communities (ARIC) Study. Nineteen significant common variant-metabolite associations were identified, including 13 novel loci (p<1.6×10^−10^). These loci were associated with 7–50% of the difference in metabolite levels per allele, and the variance explained ranged from 4% to 20%. Fourteen genes were identified within the nineteen loci, and four of them contained non-synonymous substitutions in four enzyme-encoding genes (*KLKB1*, *SIAE*, *CPS1*, and *NAT8*); the other significant loci consist of eight other enzyme-encoding genes (*ACE*, *GATM*, *ACY3*, *ACSM2B*, *THEM4*, *ADH4*, *UGT1A*, *TREH*), a transporter gene (*SLC6A13*) and a polycystin protein gene (*PKD2L1*). In addition, four potential disease-associated paths were identified, including two direct longitudinal predictive relationships: *NAT8* with N-acetylornithine, N-acetyl-1-methylhistidine and incident chronic kidney disease, and *TREH* with trehalose and incident diabetes. These results highlight the value of using endophenotypes proximal to gene function to discover new insights into biology and disease pathology.

## Author Summary

Most contemporary GWAS studies have achieved increased power by increasing the size of the discovery sample to tens of thousands of individuals. An alternative approach for detecting the effects of novel loci is to measure phenotypes that more immediately reflect the effects of gene function. The metabolome consists of a collection of small molecules resulting from a variety of cellular and biologic processes, which can be considered intermediate phenotypes proximal to gene function. Here, we report a genome-wide association study identifying nineteen genetic loci influencing untargeted metabolomes traits among African Americans in the Atherosclerosis Risk in Communities (ARIC) Study. Fourteen genes mapped within nineteen loci, including twelve enzyme-encoding genes (*KLKB1*, *SIAE*, *CPS1*, *NAT8*, *ACE*, *GATM*, *ACY3*, *ACSM2B*, *THEM4*, *ADH4*, *UGT1A* and *TREH*), a transporter gene (*SLC6A13*) and a polycystin protein gene (*PKD2L1*). In addition, four potential disease-associated paths were identified, including two direct longitudinal predictive relationships: *NAT8* with N-acetylornithine, N-acetyl-1-methylhistidine and incident chronic kidney disease, and *TREH* with trehalose and incident diabetes. These results highlight the value of using phenotypes proximal to gene function to promote novel gene discovery.

## Introduction

The power to detect genetic effects for complex traits is influenced by, among other things, the study sample size and the effect size of a particular locus. Most contemporary genome-wide association studies (GWAS) have achieved increased power by increasing the size of the discovery sample to tens of thousands of individuals [1]. Besides expanding the sample size, focusing on variants with large effects is an alternative strategy for novel gene discovery. The human metabolome consists of a collection of small molecules resulting from a variety of cellular and biologic processes, the activity of which is regulated by coordinated enzyme action [2]. In addition, as metabolites reflect multiple metabolic and physiological activities in the body, they hold promise to discover intermediate traits between gene action and disease processes [3].

GWASs of known risk factor phenotypes of clinical disease, such as cholesterol or urate levels, have shown that genetic association with functional intermediate traits, as opposed to the clinical endpoint itself, are often more highly powered and may provide information into the biological mechanism of disease [4]–[7]. Untargeted metabolomic approaches simultaneously measure numerous known and unknown metabolites present in a study sample. Recent studies combining genetics and metabolomics have identified multiple common variant-metabolite associations with large effect sizes in populations of European ancestry, and provided new functional insights into common complex disease. [8]–[11]. African ancestry-derived populations have higher levels of genetic variation and population substructure, and lower levels of linkage disequilibrium (LD) compared to European ancestry-derived populations, so studies in African-Americans may lead to identification of new genes or variants and fine map of existing loci [12]–[14]. To date, no such study has been conducted in African Americans, a population that bears a disproportionate burden of disease, such as cardiovascular disease, diabetes and chronic kidney disease [15]–[17]. Our goal here is to identify common genetic variations influencing the human metabolome in African Americans among the Atherosclerosis Risk in Communities (ARIC) Study in order to reveal novel pathways underlying disease etiology and possible avenues of disease prevention and treatment.

## Results

A total of 308 known serum metabolites including 83 amino acids, 16 carbohydrates, 9 cofactors and vitamins, 7 energies, 136 lipids, 12 nucleotides, 25 peptides and 20 xenobiotics (**Table S1**) were included and a set of 2,341,704 common autosomal SNPs were tested in 1,260 African Americans (demographics in **Table S2**) for each metabolite levels. Nineteen significant (p-value<1.6×10^−10^ after correction for multiple testing) common variant-metabolite associations were identified (locus association summaries are presented in **Table 1**, regional association plots and quantile-quantile plots are presented in **Figures S1** and **S2**, respectively), including 13 novel loci which have not been reported in previous metabolomics studies. Depending on the particular metabolite, these loci were associated with 7–50% of the difference in metabolite levels per allele (average at 25%), and the variance explained ranged from 4% to 20%.

**Table 1:** Nineteen significant GWAS loci for the human metabolome identified among African Americans in ARIC.

Metabolites	Top SNP	Ref Alleles	Minor Allele	MAF	P	Gene	Gene function	Published GWAS phenotypes of the gene
[H]HWESASLLR[OH]	rs4343	A/G	G	0.25	1×10^−18^	*ACE* (synonymous)	Angiotensin I converting enzyme, a dipeptidyl carboxypeptidase	aspartyphenylalanine, angiotensin-converting enzyme activity
Aspartylphenylalanine	rs4343	A/G	G	0.25	9×10^−25^	*ACE* (synonymous)	Angiotensin I converting enzyme, a dipeptidyl carboxypeptidase	aspartyphenylalanine, angiotensin-converting enzyme activity
HXGXA	rs3733402	A/G	G	0.26	9×10^−27^	*KLKB1* (missense)	Kallikrein B, plasma 1, targeted action of bradykinin	bradykinin, his/val, response to statin therapy, endothelin-1, adrenomedullin, IGF-1
Threonylphenylalanine	rs4363	A/G	G	0.42	8×10^−14^	*ACE* (intron)	Angiotensin I converting enzyme, a dipeptidyl carboxypeptidase	aspartyphenylalanine, angiotensin-converting enzyme activity
Creatine	rs2433610	C/T	T	0.49	9×10^−12^	15 kb from *GATM*	Glycine amidinotransferase, an enzyme involved in creatine biosynthesis	renal function, chronoic kidney disease
Glycine	rs7422339	A/T	A	0.32	4×10^−12^	*CPS1* (missense)	Carbamoyl-phosphate synthase 1, catalyze the synthesis of carbamoyl phosphate to produce glycine	glycine, eGFRcrea, homocysteine levels, fibrinogen, BMI, non-small cell lung cancer
N-acetylornithine	rs13538	A/G	A	0.48	4×10^−66^	*NAT8* (missense)	N-acetyltransferase 8	N-acetylornithine, creatinine levels, chronic kidney disease, glomerular filtration rate
N-acetylphenylalanine	rs12288023	C/T	C	0.09	9×10^−16^	3 kb from *ACY3*	Aspartoacylase 3, a hydrolase that removes the acyl group from several acylated aromatic amino acids, such as N-acetyl-L-phenylalanine	/
Phenylacetate	rs7499271	A/T	A	0.25	6×10^−11^	*ACSM2B* (intron)	Acyl-CoA synthetase medium-chain family member 2B, phenylacetate is used as its substrate	/
3-hydroxydecanoate	rs10788817	C/G	C	0.46	3×10^−13^	0.7 kb from *THEM4*	Thioesterase superfamily member 4, a major downstream target of receptor tyrosine kinases	/
Acetylcarnitine	rs12282107	C/T	C	0.24	5×10^−14^	*SIAE* (missense)	Sialic acid acetylesterase, possess sialic acid 9-O-acetylesterase activity	/
Deoxycarnitine	rs555044	A/C	A	0.43	1×10^−12^	*SLC6A13* (intron)	Solute carrier family 6 member 13, mediate the removal of neurotransmitter transport and maintain extracellcular levels	chronic kidney disease
Hexadecanedioate	rs17028615	A/G	G	0.23	2×10^−15^	6 kb from *ADH4*	Alcohol dehydrogenase 4, proliferating cell nuclear antigen pseudogene 1	esophageal cancer
Palmitoleate (16:1n7)	rs603424	A/G	G	0.32	1×10^−11^	*PKD2L1* (intron)	Polycystic kidney disease 2-like 1 protein	palmitic acid (16:0), phospholipid levels, total antioxidants
Leucylphenylalanine	rs3733402	A/G	G	0.26	7×10^−25^	*KLKB1* (missense)	Kallikrein B, plasma 1, targeted action of bradykinin	bradykinin, his/val, response to statin therapy, endothelin-1, adrenomedullin, IGF-1
Bilirubin (E,E)	rs887829	C/T	T	0.44	1×10^−17^	*UGT1A* (intron)	UDP glucuronosyltransferase 1 family, polypeptide A complex locus, with bilirubin as its preferred substrate	bbilirubin levels, bladder cancer
Bilirubin (Z,Z)	rs887829	C/T	T	0.44	6×10^−13^	*UGT1A* (intron)	UDP glucuronosyltransferase 1 family, polypeptide A complex locus, with bilirubin as its preferred substrate	bilirubin levels, bladder cancer
Biliverdin	rs887829	C/T	T	0.44	8×10^−23^	*UGT1A* (intron)	UDP glucuronosyltransferase 1 family, polypeptide A complex locus, with bilirubin as its preferred substrate	bilirubin levels, bladder cancer
Trehalose	rs507080	A/G	A	0.35	3×10^−30^	*TREH* (intron)	Trehalase, uses trehalose as the only substrate	height

Top SNP indicates the SNP with the lowest p-value; Ref Alleles, coded/non-coded alleles; MAF, minor allele frequency. All metabolites values were natural log-transformed prior to the analyses.

**Figure 1:**
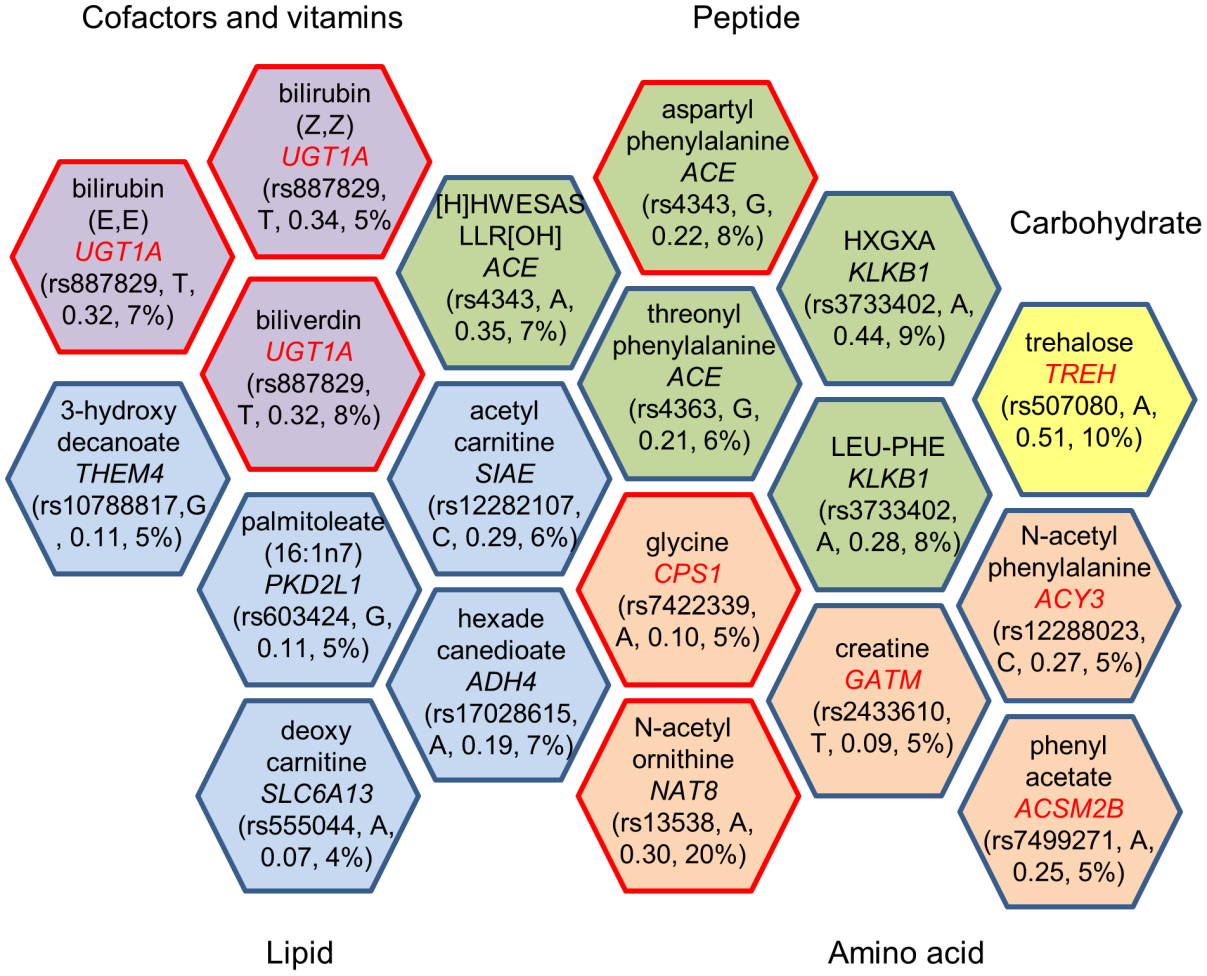
Genome-wide significant loci and human metabolic traits among African Americans in ARIC. Each hexagon shows the significant genetic locus (p<1.6×10^−10^) and the corresponding metabolite. The gene name listed in a hexagon is mapped by the sentinel SNP, and the closest gene is picked if the sentinel SNP was not located in a gene but is in linkage disequilibrium (r^2^≥0.8) with other SNPs in a nearby gene. Metabolites are grouped by super pathway, indicated in different colors. A red border line indicates that this gene-metabolite pair has been previously reported, and a gene name in red indicates the gene encodes an enzyme that catalyzes the reaction of the corresponding metabolite as a substrate or product. Rs number, risk allele, effect size and variance explained for the sentinel SNP are listed in parenthesis.

Fourteen genes were mapped within the nineteen significant genetic loci; eight of them encode enzymes that catalyze the reaction of the corresponding metabolite as a substrate or product (gene names shown in red in **Figure 1**). Four of the associated loci contained non-synonymous substitutions in four enzyme-encoding genes (*KLKB1*, *SIAE*, *CPS1*, and *NAT8*). The other significant loci consist of eight other enzyme-encoding genes (*ACE*, *GATM*, *ACY3*, *ACSM2B*, *THEM4*, *ADH4*, *UGT1A*, and *TREH*), a transporter gene (*SLC6A13*) and a polycystin protein gene (*PKD2L1*). Two protease-encoding genes, *ACE* and *KLKB1*, showed pleiotropic effects on multiple oligopeptide metabolites, and the UDP-glucuronosyltransferases gene, *UGT1A*, contributed to the levels of several bile pigments (**Figure 1**).

Nineteen significant common variant-metabolite associations were compared with previously published SNP-metabolite associations in Caucasians [10]. Eleven out of nineteen metabolites were shared between the published study and the data presented here, and six of them showed the same significant SNP-metabolite associations in both ethnicities (**Table 2**). A *CPS1*-glycine association was reported in the Caucasion metabolomic GWAS, but the sentinel SNP was different (r^2^<0.5) from that reported here (**Table 2**). A *CPS1*-glycine association was also reported in a recent genetic study for glycine metabolism among Caucasians [18]. The other four shared metabolites had different signals in African-Americans when compared to Caucasians (**Table S3**).

**Table 2:** A comparison of significant common variant-metabolite association among ARIC, KORA and TwinsUK studies.

Metabolites	ARIC		KORA		TwinsUK	
	Top SNP	P	SNP	P	SNP	P
aspartylphenylalanine	rs4343 *ACE* (synonymous)	9×10^−25^	rs4343	2×10^−10^	rs4343	2×10^−10^
N-acetylornithine	rs13538 *NAT8* (missense)	4×10^−66^	rs6745480 (r^2^ = 1)	3×10^−123^	rs10496191 (r^2^ = 0.95)	2×10^−65^
palmitoleate (16:1n7)	rs603424 *PKD2L1* (intron)	1×10^−11^	rs603424	1×10^−7^	-	-
bilirubin (E,E)	rs887829 *UGT1A* (intron)	1×10^−17^	rs887829	3×10^−24^	rs887829	5×10^−5^
bilirubin (Z,Z)	rs887829 *UGT1A* (intron)	6×10^−13^	rs887829	1×10^−46^	rs887829	4×10^−8^
biliverdin	rs887829 *UGT1A* (intron)	8×10^−23^	rs887829	5×10^−47^	-	-
glycine	rs7422339 *CPS1* (missense)	4×10^−12^	rs2371015 (r^2^<0.5)	3×10^−9^	rs4673553 (r^2^<0.5)	2×10^−23^

We identified a missense mutation in *NAT8* (rs13538) that was significantly associated with N-acetylornithine levels (p = 4.0×10^−66^). A recent biochemical study has shown that *NAT8* catalyzed the N-acetylation of cysteine conjugates [19]. We next asked whether the presumed specificity of *NAT8*'s function could be used to identify the identity of any unknown metabolites by analyzing its effect on 294 unknown metabolites. Two metabolites, X-11333 and X-11787 reached our *a priori* defined level of significance (p = 1.0×10^−61^ and p = 2.5×10^−25^, respectively). By targeted mass spectroscopy, X-11333 was determined to be N-acetyl-1-methylhistidine (**Figure S3**), a type of N-acetyl amino acid; and X-11787 was an isoform of either hydroxy leucine or isoleucine, as reported previously [20].

**Figure 2:**
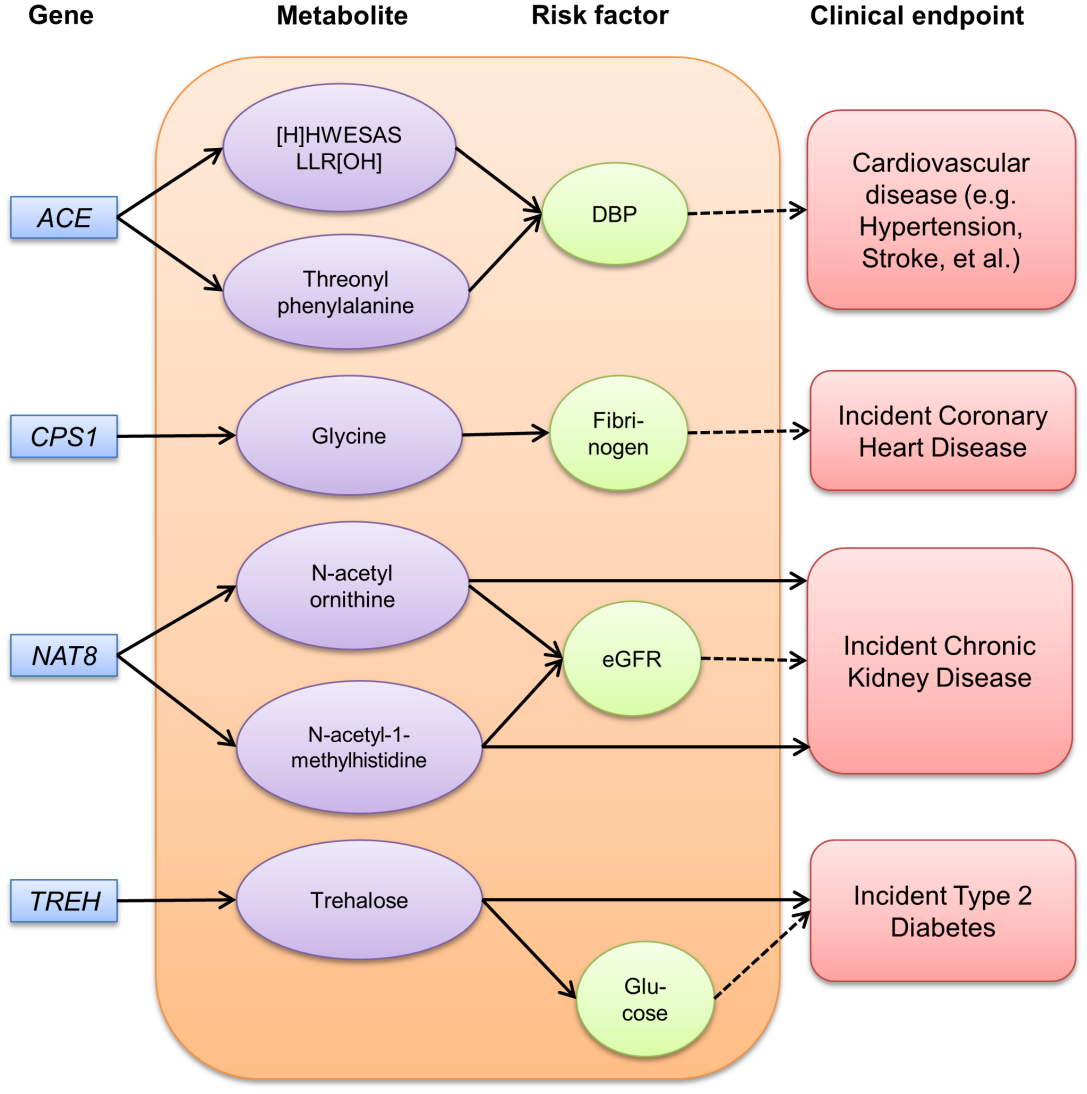
Pathways among gene, metabolite, risk factor and disease identified among ARIC African Americans. Solid arrows between genes and metabolites indicate genome-wide significant effects (p<1.6×10^−10^). Arrows between metabolites and risk factors indicate significant linear associations after adjusting for age and gender (p<0.05). Arrows between metabolites and clinical endpoint indicate significant associations after adjusting for age, gender and other risk factors using Cox proportional hazards modeling (p<0.05). The dotted arrows between risk factors and clinical endpoints indicate well-established relationships. DBP indicates diastolic blood pressure and eGFR, estimated glomerular filtration rate.

Among nineteen metabolites that reached genome-wide significance, we identified four potential disease-associated paths among African Americans for cardiovascular disease, chronic kidney disease (CKD) and diabetes, including two direct longitudinal associations (**Figure 2**, detailed estimates in **Table S4**). As described above, a missense mutation in *NAT8* (rs13538), a known susceptibility locus for chronic kidney disease [21], was significantly associated with N-acetylornithine and N-acetyl-1-methylhistidine levels. We identified a pronounced relationship of both N-acetylornithine and N-acetyl-1-methylhistidine levels with kidney function, whereby higher levels of of N-acetylornithine and N-acetyl-1-methylhistidine were related to lower eGFR (p = 9.0×10^−13^ and 1.6×10^−21^; respectively) and higher risk of incident CKD after 19 average years of follow-up among 1,921 African Americans (demographics in **Table S5**, HR = 1.64, p = 0.003 and HR = 1.34, p = 0.03, respectively). However, the longitudinal associations with the metabolites were attenuated and no longer significant after further adjusting for eGFR (data not shown). Finally, trehalose levels were significantly associated with *TREH* gene variation. Trehalose can be cleaved to two molecules of glucose. In this study, trehalose levels were significantly associated with glucose levels (p = 2.9×10^−17^), and showed a 1.34 fold increased risk of incident diabetes after an average 7 years of follow-up (p = 2.0×10^−5^) in a sample of 1,430 ARIC African Americans (demographics in **Table S5)**. With further adjustment of glucose levels, trehalose levels persisted to show an apparent association with incident diabetes, although the effect size was lessened (HR = 1.16, p = 0.02).

## Discussion

By combining high-throughput metabolomic and genomic technologies, we identified nineteen common variant-metabolite associations among African Americans with p-values ranging from 6.0×10^−11^ to 4.0×10^−66^. We inferred the structure of an unknown metabolite to be N-acetyl-1-methylhistidine using knowledge of the associated gene's function and targeted mass spectroscopy. We further established potential novel disease-associated pathways for cardiovascular disease risk factors, CKD and diabetes. The results offer new evidence about the genetic impact on metabolites and disease among African Americans, which advance our understanding of disease causation and progression.

Most loci identified by GWA studies of complex disease traits contribute relatively small effects and the variance explained remains modest [14], [22], [23]. Thus, contemporary GWAS are shifting focus to phenotypes that more immediately reflect the effects of gene action. For example, although the effect sizes of genetic loci related to coronary heart disease (CHD) are relatively small (OR from 1.08 to 1.47) [24]–[26], loci related to plasma triglyceride and cholesterol levels explained a meaningful proportion of the variance (9–13%) [4]. The human metabolome, the ultimate downstream product of gene and environment interaction, holds the promise to identify genes that directly reflect gene action with large effects sizes [8], [10], [27]. Our results show relatively large effect sizes of nineteen identified genetic loci related to human metabolome among African Americans (average at 25% shift per allele copy). In addition, the majority of identified loci (15/19) are located in or near genes, and these loci explained up to 20% of the variance of each trait.

Twelve out of fourteen genes that were significantly associated with metabolite levels were enzyme-encoding genes, including four genes involved in disease-associated processes. These data underscore the important role of enzyme activity and regulation in controlling metabolite levels. As metabolite levels are closely related to disease process, to understand whether the underlying mutations detected here lead to gain-of-function or loss-of-function for these enzyme-encoding genes offers new opportunities for disease treatment and prevention (e.g. design an antagonist/agonist of the gene as a drug candidate). The majority of the gene-metabolite associations are consistent with the gene's known function, but the direction of effect of the coded allele does not provide direct evidence as to whether or not the variant represents gain of function or loss of function. Future investigation of the functional impact of the underlying causal variants is critical and is an area of intense research.

*NAT8* is expressed mainly in the kidney and liver [28], but its function is not fully understood. Several previous, seemingly unrelated, observations have found that mutations in N-acetyltransferase 8 (*NAT8*), are contributed to N-acetylornithine levels, creatinine levels, kidney function and CKD [10], [21], [29], [30]. Our results show that an amino acid substitution in *NAT8* is related to N-acetylornithine, N-acetyl-1-methylhistidine and eGFR, which in-turn influence risk to incident CKD. These findings provide evidences that N-acetylation plays a role in the development of CKD [10].

Trehalose is a food ingredient with the ability to prevent protein denaturation [31]. Because of its ability to inhibit lipid and protein misfolding, trehalose has become a potential therapeutic in neurodegenerative studies [32], [33]. Animal safety studies concluded that trehalose is safe for use as an ingredient in consumer products [34], and it is now widely used in food and cosmetics. Here, we report that trehalose levels are regulated by *TREH*, which encodes the trehalase enzyme which hydrolyzes trehalose to two glucose molecules. In addition, we show that trehalose is associated with glucose levels and the onset of incident diabetes.

Environment factors, in addition to and interacting with genetic factors, (e.g. dietary intake) explain part of the variability of human metabolome. Follow-up investigations of the interactions between the genes identified here and possible environment factors are likely to provide new insight into the understanding of disease etiology and its metabolism. For example, alcohol dehydrogenase 4 (*ADH4*) contributes to esophageal squamous-cell carcinoma (ESCC) through an interaction with alcohol consumption [35]. Here, we reported that *ADH4* is associated with hexadecanedioate levels, a metabolite with an antitumor activity [36]. Moreover, studies have shown that coffee consumption is associated with lower bilirubin levels [37] and *UGT1A* is contributed to bilirubin levels as well [10]. Our data show that mutations in *UGT1A* are associated with the levels of several bile pigments. Thus, future investigations of genes related to metabolite levels with environment interaction are of interest.

Untargeted metabolomics approaches measure numerous known and unknown metabolites presented in a sample simultaneously. Since the chemical identities for unknown metabolites have not been elucidated, previous GWAS on metabolomic traits largely ignored unknown metabolites for the analysis. In our study, we show an example of unknown metabolite identification (i.e. X-11333) by combining GWAS results (i.e. *NAT8*) with existing knowledge about the function of the gene product (i.e. N- acetylation). A recent study has used GWAS results and Gaussian graphical modeling to predict unknown metabolite identities [38]. These two examples demonstrate the feasibility for unknown compounds structure identification by combing genetic and metabolomics information.

Limitation of this study warrants consideration. To our knowledge, the ARIC study is the only cohort with serum metabolome measurements in African-Americans, so it is unlikely to find an independent sample for replication. In our study, the SNP-metabolite associations identified were compared with the results from a published study in Caucasians [10] as a surrogate replication. Six distinct SNP-metabolite associations were replicated out of eleven shared metabolites, indicating homogeneous genetic effects on several metabolites regardless of ethnicities. Differences in the site frequency spectrum between African-Americans and Caucasians and lower LD in African-Americans may explain the lack of significant association at the other loci. As a consequence of lack of replication, the proportion of variance explained by the SNPs was reported from the discovery sample, which may be an over-estimate. Future studies are needed to replicate our findings in independent samples of African-Americans. Despite limitations, the data presented here have important strength. Previously published GWAS on human metabolites estimate only cross-sectional relationships between metabolites and clinical endpoints. In contrast, the data presented here originate from a large, well-defined, longitudinal cohort study, allowing establishments of longitudinal predictive relationships.

In summary, we report here the first genome-wide association study of untargeted metabolome in African-Americans. The genetic variant-metabolite associations along with the disease path reported here will continue to be improved with further use of contemporary omics technologies. Our study highlights the value of utilizing omics studies in deeply phenotyped individuals to provide new insights into gene function, disease etiology and epidemiology.

## Methods

### Study Population

The Atherosclerosis Risk in Communities (ARIC) study is a longitudinal cohort study designed to ascertain the etiology and predictors of cardiovascular disease (CVD). The ARIC study enrolled 15,792 middle-aged adults from four U.S. communities (Forsyth County, NC; Jackson, MS; suburbs of Minneapolis, MN; and Washington County, MD) between 1987–89 and followed by four completed visits with each approximately three years apart, in 1987–89, 1990–92, 1993–95, and 1996–98. In general, each visit included interviews and a physical examination. A detailed description of the ARIC study design and methods was published elsewhere [39]. Metabolomic profiles were measured in baseline serum from 1,977 African-Americans selected from the Jackson, MS field center. Participants were excluded if they did not give consent for use of DNA information.

### Assessment of Metabolomic Profiles

Metabolite profiling was completed in June 2010 using fasting serum samples which had been stored at −80° since collection at the baseline examination in 1987–1989. In total, detection and quantification of 602 metabolites was completed by Metabolon Inc. (Durham, USA) using an untargeted, gas chromatography-mass spectrometry and liquid chromatography-mass spectrometry (GC-MS and LC-MS)-based metabolomic quantification protocol [40], [41]. Prior to the analyses presented here, a rigorous assessment of the metabolomic data was done. Metabolites were excluded if: 1) more than 50% of the samples had values below the detection limit; or 2) they had unknown chemical structures, except for X-11333 and X-11787 which were followed-up as part of more detailed *NAT8* investigations. After this assessment, a total of 308 named metabolites were included in the present study. Structural identifications for X-11333 and X-11787 were proposed using a mass spec-based structural approach, including targeted accurate mass and MS^n^ fragmentation with accurate mass [41].

### Genotyping and Imputation

In the present study, common (minor allele frequency, MAF≥5%) autosomal single-nucleotide polymorphisms (SNPs) were genotyped on the Affymetrix 6.0 chip and were imputed to 2,341,704 SNPs based on a panel of cosmopolitan reference haplotypes from HapMap CEU and YRI. MACH v1.0 was used to do imputation and allele dosage information was summarized in the imputation results. SNPs were excluded before imputation if they had no chromosomal location, were monomorphic, had a call rate <95%, or had a Hardy-Weinberg equilibrium p-value<10^−5^. For each SNP, the ratio of the observed versus expected variance of the dosage served as a measure of imputation quality.

### Genome-Wide Association Analyses

A total of 308 metabolites were included in this analysis. Metabolite levels below the detectable limit of the assay were imputed with the lowest detected value for that metabolite in all samples, and all metabolites values were natural log-transformed prior to the analyses. Linear regressions and an additive genetic model were applied to each metabolite, adjusting for age, sex and the first 10 principal components. The significant threshold was defined as a p-value<1.6×10^−10^ (5.0×10^−8^/308) based on Bonferroni correction. SNPs with MAF<5% were excluded. Quantile-quantile (QQ) plots were generated for each analysis to illustrate the distribution of the observed and expected p-values for all eligible SNPs. Regional plots showing LD and the location of nearby genes (if any) were generated for the top ranking SNPs for each metabolite. If more than one significant SNP clustered at a locus, the SNP with the smallest p-value was reported as the sentinel marker. All analyses were performed using ProbABEL and R (www.r-project.org). The identified sentinel SNPs were further compared with the metabolite-SNP association from the KORA and TwinsUK studies [10] using their public GWAS server (http://metabolomics.helmholtz-muenchen.de/gwa/index.html) and other published GWA studies through NHGRI GWAS Catalog (http://www.genome.gov/gwastudies/).

### Disease Association Analyses

Analyses included all African-American samples with metabolomic data were conducted to estimate the association between genome-wide significant metabolite levels and relevant clinical risk factors and endpoints, including incident chronic kidney disease and incident type 2 diabetes. Nine associations, including six cross-sectional associations with clinical risk factors and three longitudinal associations with clinical endpoints, were tested. In each analysis, metabolite levels were natural log-transformed. The cross-sectional associations were assessed using linear regression with adjustment for age and gender. Longitudinal associations with disease endpoints were estimated using Cox proportional hazards models adjusting for age, gender, systolic blood pressure (SBP), antihypertensive medication use, diabetes, high-density lipoprotein, low-density lipoprotein, current smoking and prevalent CHD for incident the CKD analysis; and age, gender, SBP, antihypertensive medication use, body mass index, total cholesterol for the incident type 2 diabetes analysis. The proportional hazards assumption was examined and not rejected using the methods developed by Grambsch and Therneau [42]. Covariates were measured at baseline (1987–1989) and The Chronic Kidney Disease Epidemiology Collaboration equation was applied to estimate glomerular filtration rate (eGFR_CKD-EPI_) [43]. For the disease association analyses, the significant threshold was defined as p<0.005 using Bonferroni correction (0.05/9) and the analyses were performed using R (www.r-project.org).

## Supporting Information

Figure S1 Regional association plots of the top ranking genome-wide significant markers for 19 metabolites.
(DOCX)

Figure S2 Quantile-quantile (QQ) plots of the expected and observed –log p-values for 19 metabolites.
(DOCX)

Figure S3 MS/MS fragmentation spectrum analysis of parent molecule for X-11333.
(DOCX)

Table S1 List of 308 named metabolites measured in ARIC.
(DOCX)

Table S2 Baseline characteristics of African-Americans in ARIC for genetic analyses.
(DOCX)

Table S3 A comparison of common variant-metabolite association among ARIC, KORA and TwinsUK studies.
(DOCX)

Table S4 Association between genome-wide significant metabolites and clinical endpoints among African-Americans in ARIC.
(DOCX)

Table S5 Baseline characteristics of African-Americans in ARIC for incident disease association analyses.
(DOCX)
