# Supplementary material for: Genetic Determinants Influencing Human Serum Metabolome among African Americans
Source: PLoS Genet. 2014 Mar 13;10(3):e1004212. doi: 10.1371/journal.pgen.1004212 (PMC3952826; doi:10.1371/journal.pgen.1004212)

**Figure S1.** Regional association plots of the top ranking genome-wide significant markers for 19 metabolites.


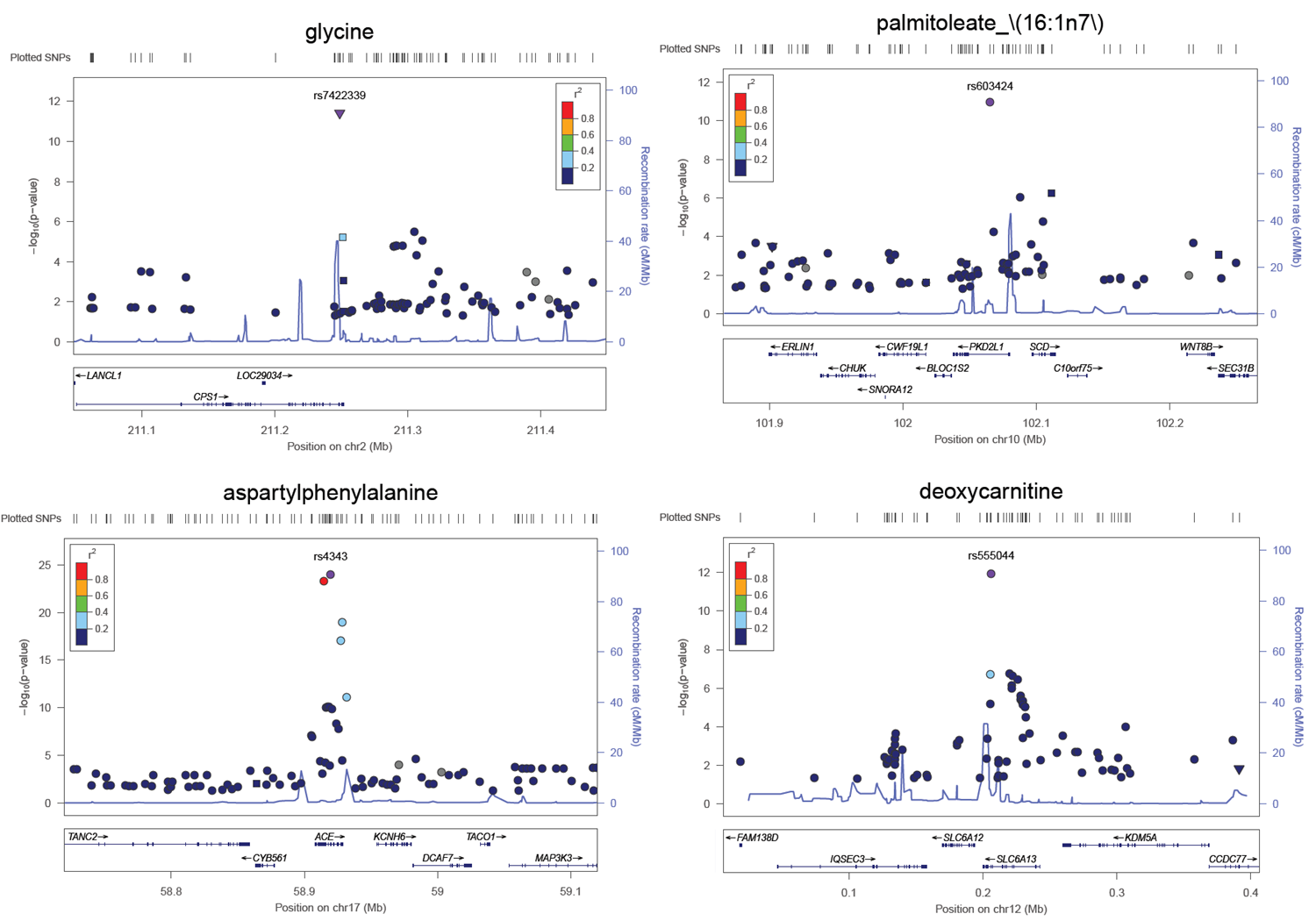


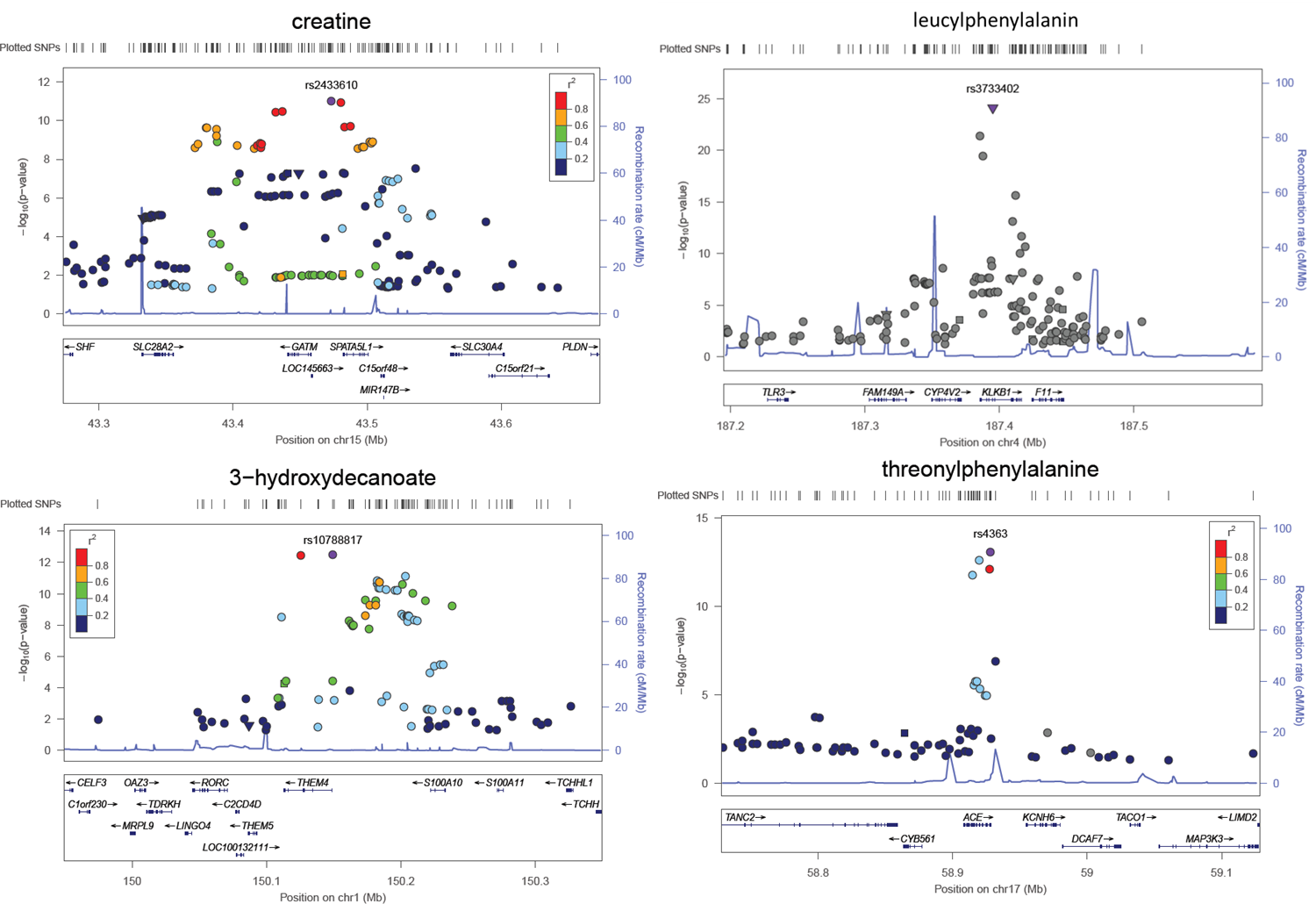


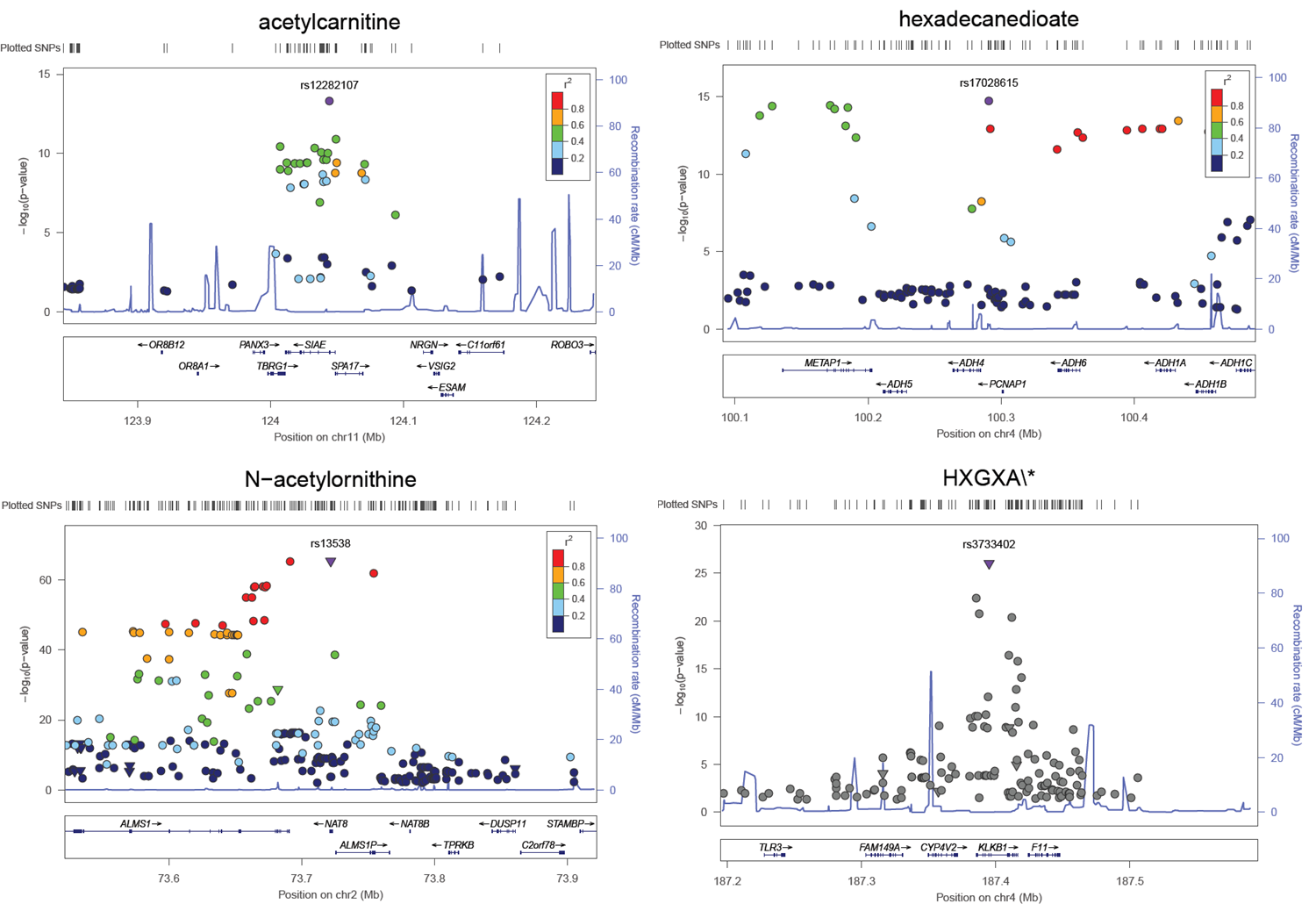


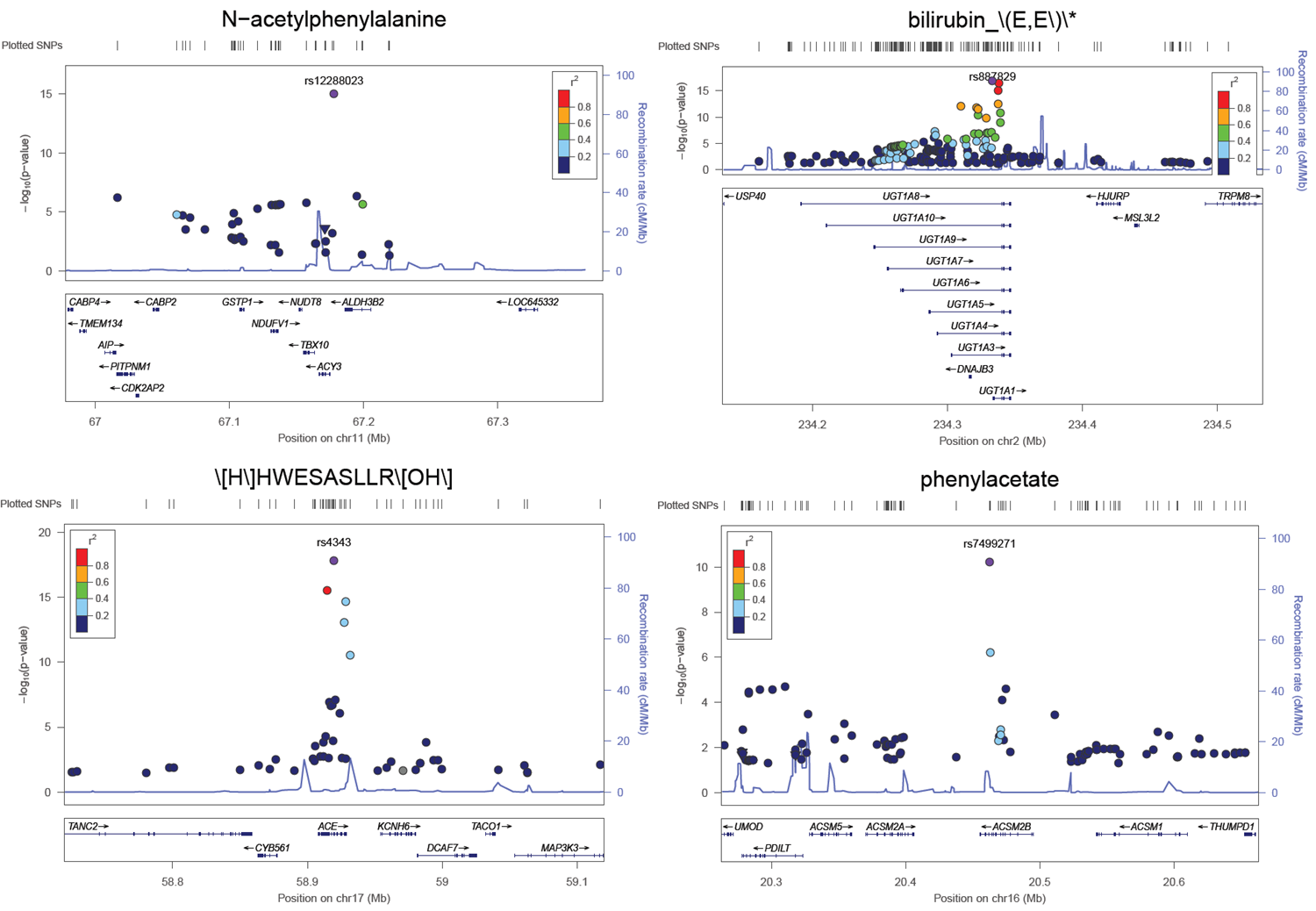


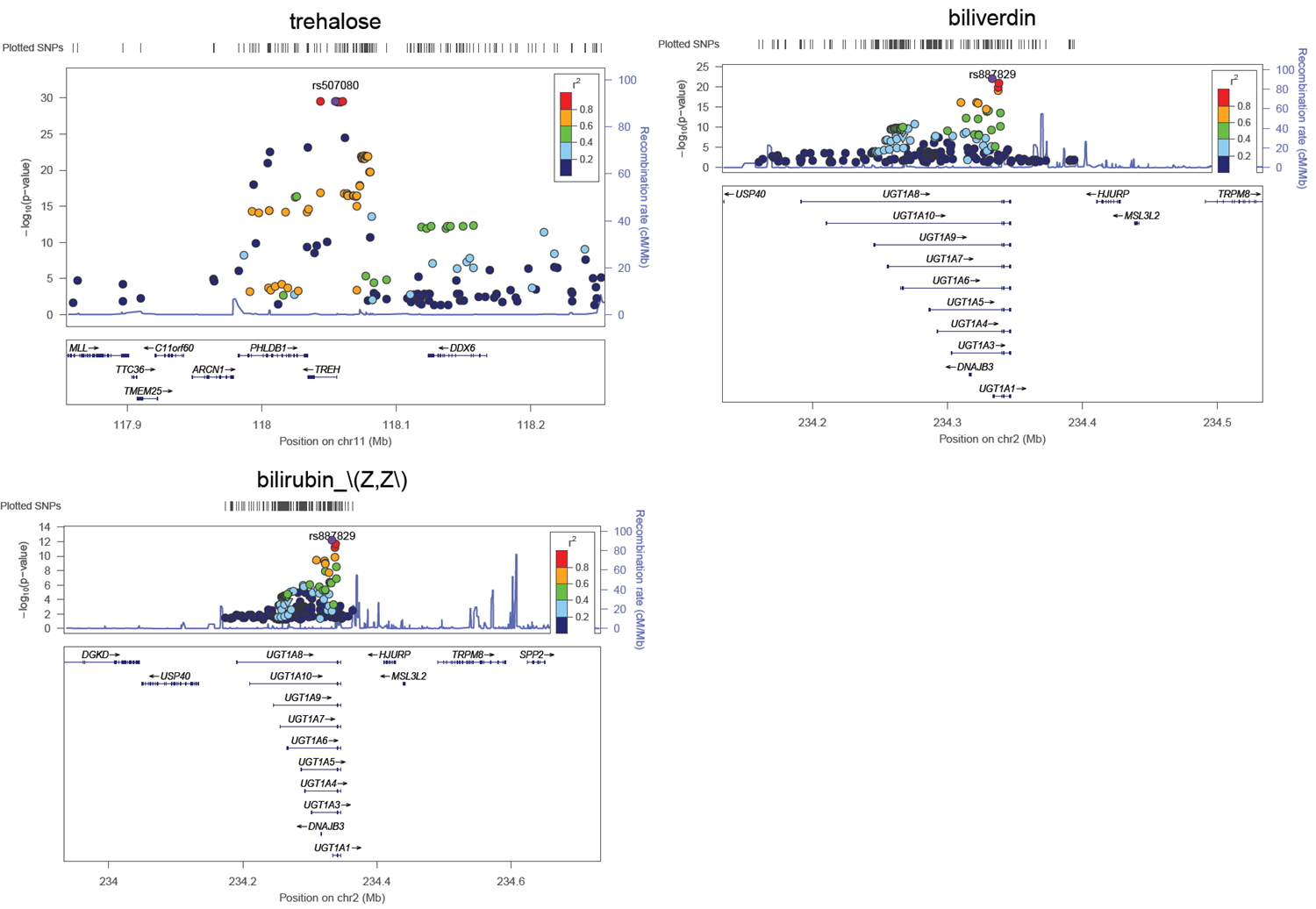

Supplement: Figure S1 — Regional association plots of the top ranking genome-wide significant markers for 19 metabolites. (DOCX) [file pgen.1004212.s001.docx]
