# Supplementary figures and images for: Genetic Determinants Influencing Human Serum Metabolome among African Americans
Source: PLoS Genet. 2014 Mar 13;10(3):e1004212. doi: 10.1371/journal.pgen.1004212 (PMC3952826; doi:10.1371/journal.pgen.1004212)

**Figure S2.** Quantile-quantile (QQ) plots of the expected and observed –log p-values for 19 metabolites.


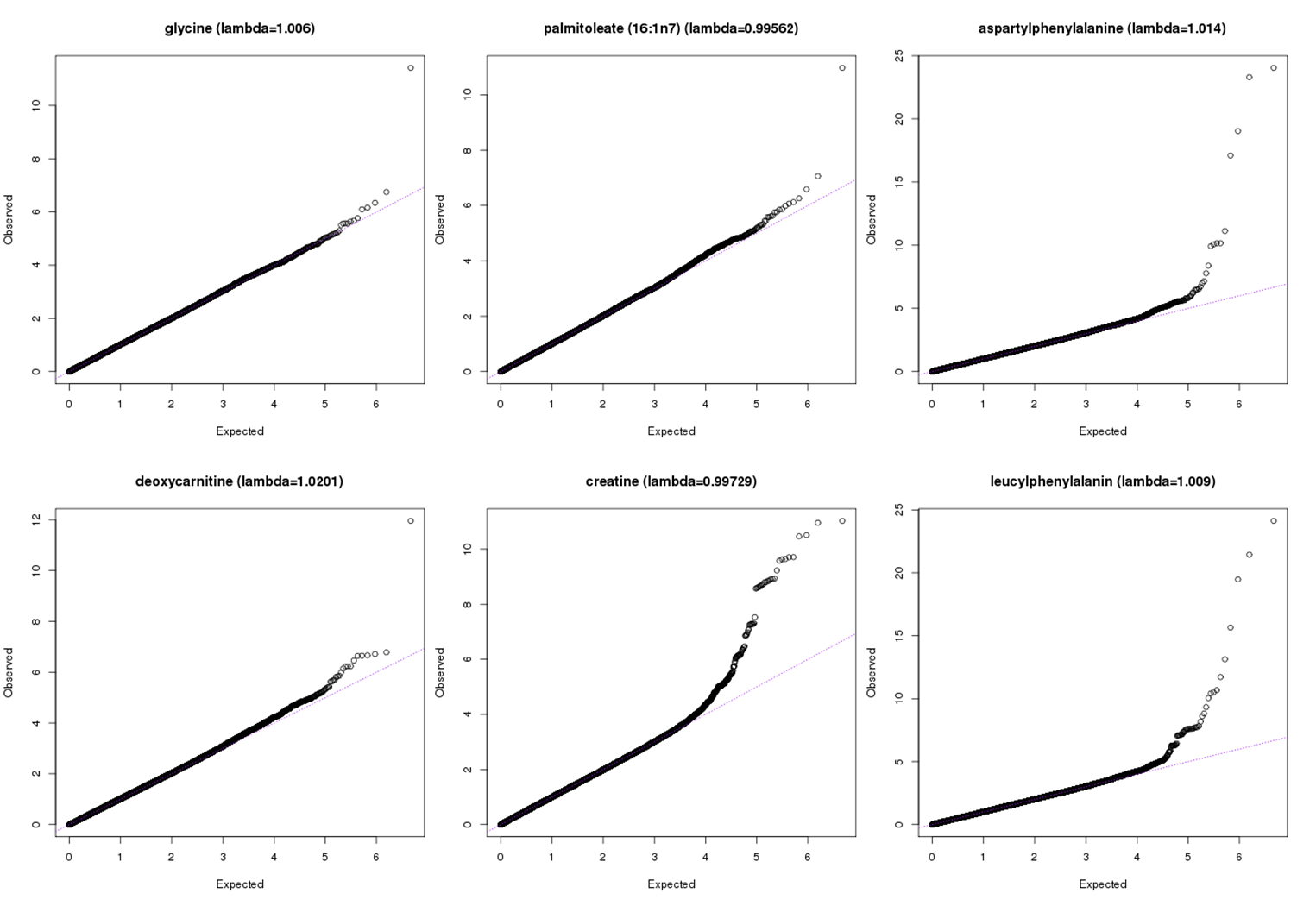


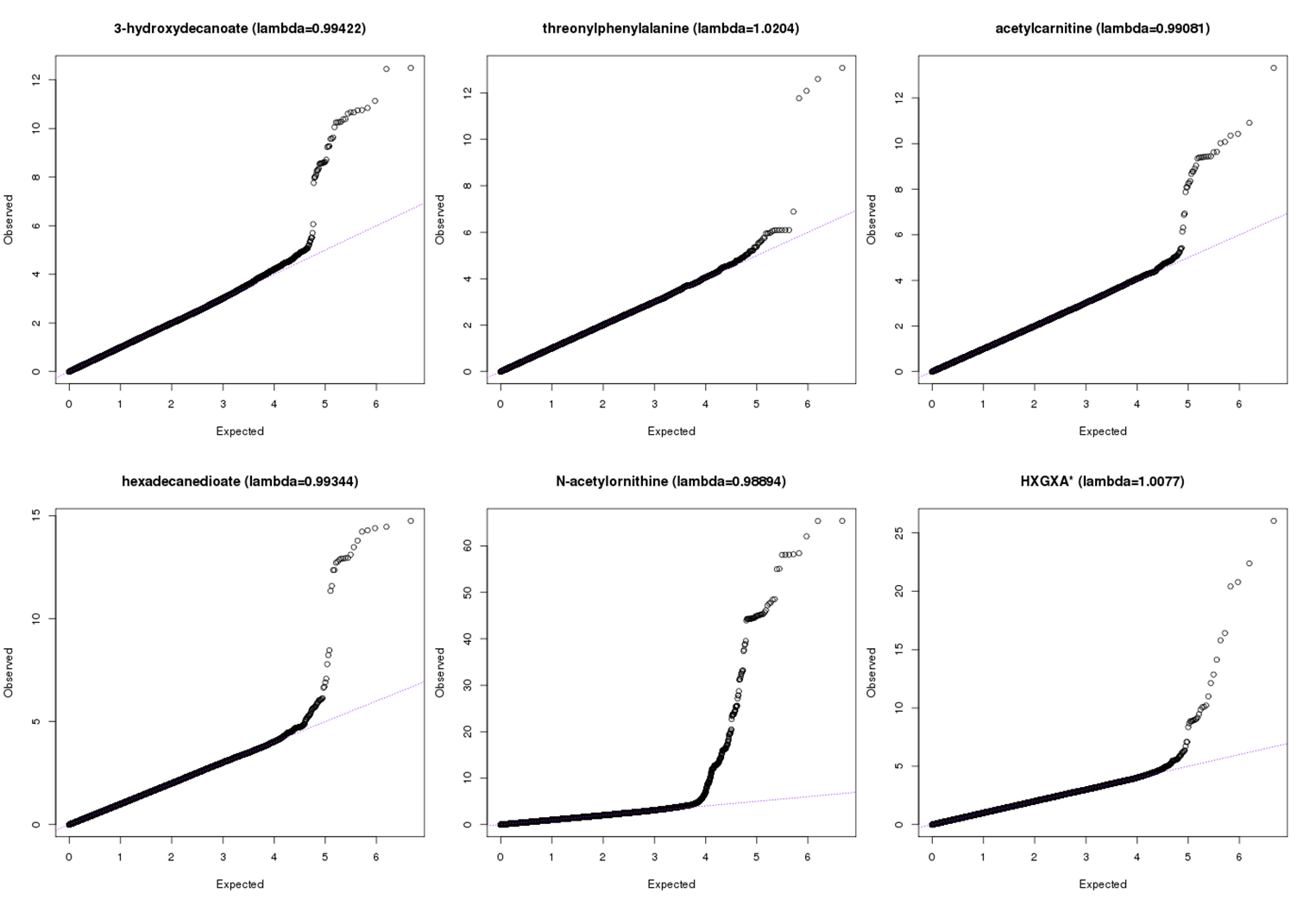


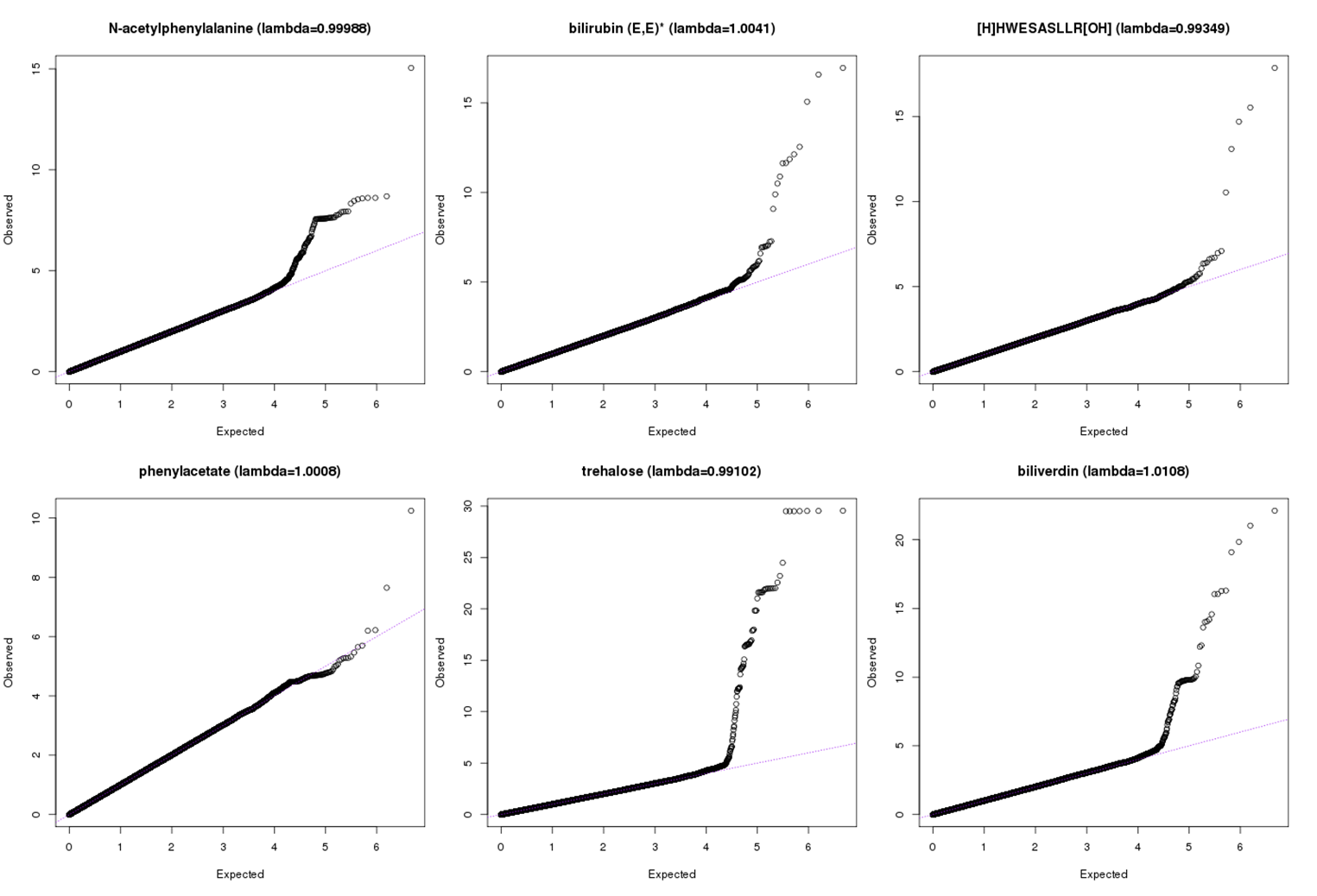


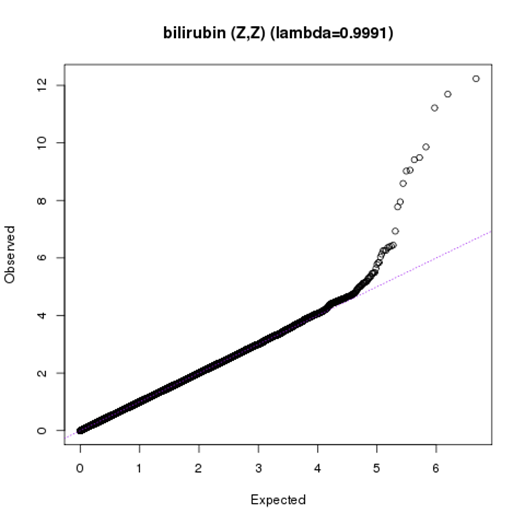

Supplement: Figure S2 — Quantile-quantile (QQ) plots of the expected and observed –log p-values for 19 metabolites. (DOCX) [file pgen.1004212.s002.docx]

**Figure S3.** MS/MS fragmentation spectrum analysis of parent molecule for X-11333


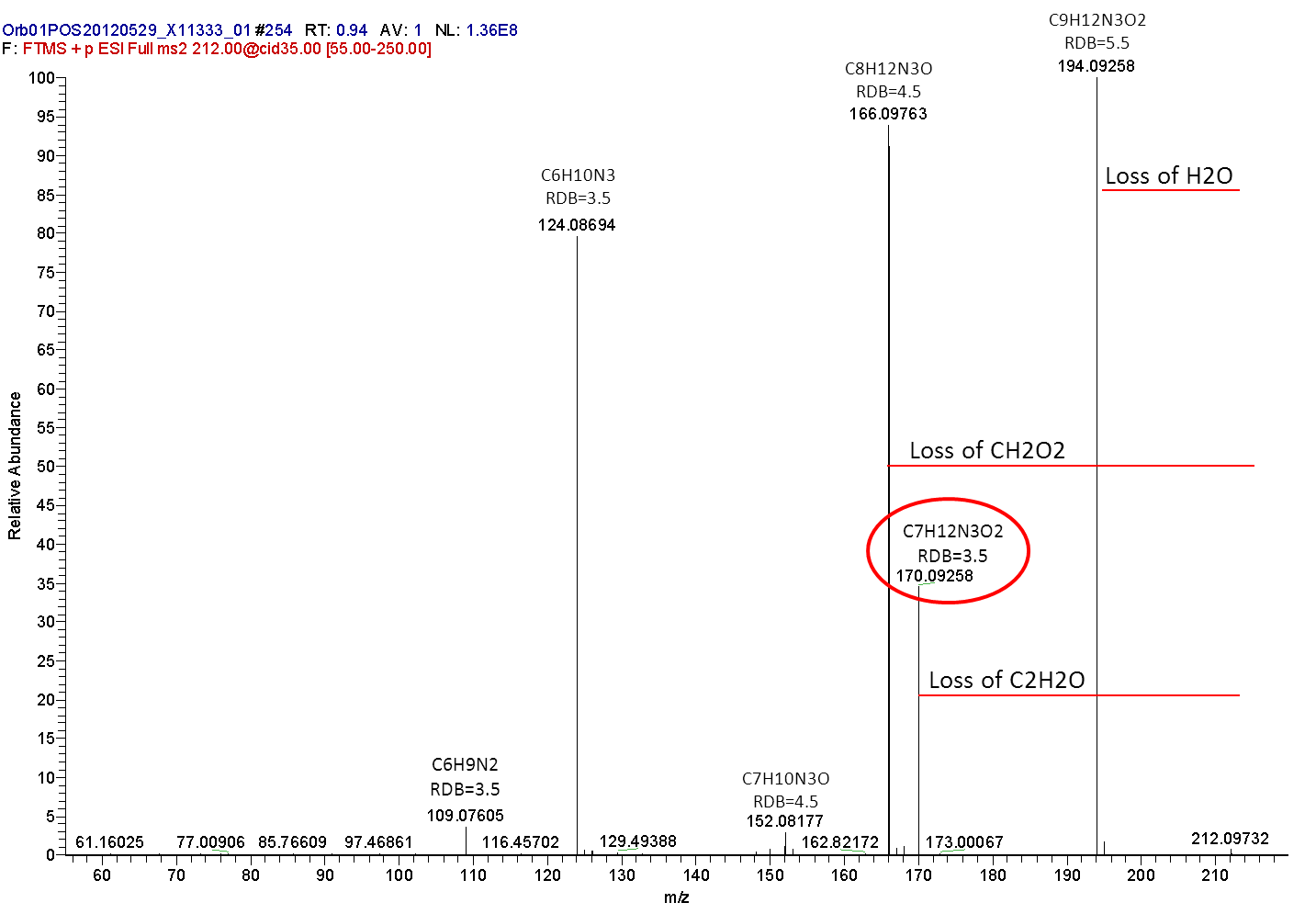

Supplement: Figure S3 — MS/MS fragmentation spectrum analysis of parent molecule for X-11333. (DOCX) [file pgen.1004212.s003.docx]
