# Supplementary material for: Genetic Determinants Influencing Human Serum Metabolome among African Americans
Source: PLoS Genet. 2014 Mar 13;10(3):e1004212. doi: 10.1371/journal.pgen.1004212 (PMC3952826; doi:10.1371/journal.pgen.1004212)
Supplement: Table S1 — List of 308 named metabolites measured in ARIC. (DOCX) [file pgen.1004212.s004.docx]

**Table S1**. Metabolites measured among African-Americans in ARIC. For each metabolite we report the super‐pathway, sub-pathway, measurement platform, iron mass and percent of low values imputed for the analyses presented here.

| Metabolite | **Super-**  **Pathway** | **Sub-Pathway** | **Plat-**  **form** | **Imputated (%)** |
| --- | --- | --- | --- | --- |
| 2-aminobutyrate | Amino acid | Butanoate metabolism | LC/MS pos | 0 |
| 2-hydroxybutyrate (AHB) | Amino acid | Cysteine, methionine, SAM, taurine metabolism | GC/MS | 0 |
| 2-hydroxyisobutyrate | Amino acid | Valine, leucine and isoleucine metabolism | GC/MS | 11.94 |
| 2-methylbutyroylcarnitine | Amino acid | Valine, leucine and isoleucine metabolism | LC/MS pos | 9.17 |
| 3-(4-hydroxyphenyl)lactate | Amino acid | Phenylalanine & tyrosine metabolism | LC/MS neg | 0 |
| 3-hydroxy-2-ethylpropionate | Amino acid | Valine, leucine and isoleucine metabolism | LC/MS neg | 30.35 |
| 3-hydroxyisobutyrate | Amino acid | Valine, leucine and isoleucine metabolism | GC/MS | 4.29 |
| 3-indoxyl sulfate | Amino acid | Tryptophan metabolism | LC/MS neg | 0 |
| 3-methoxytyrosine | Amino acid | Phenylalanine & tyrosine metabolism | LC/MS pos | 7.35 |
| 3-methyl-2-oxovalerate | Amino acid | Valine, leucine and isoleucine metabolism | LC/MS neg | 30.86 |
| 3-methylhistidine | Amino acid | Histidine metabolism | LC/MS neg | 11.86 |
| 3-phenylpropionate (hydrocinnamate) | Amino acid | Phenylalanine & tyrosine metabolism | LC/MS neg | 21.03 |
| 4-acetamidobutanoate | Amino acid | Guanidino and acetamido metabolism | LC/MS pos | 0.44 |
| 4-guanidinobutanoate | Amino acid | Guanidino and acetamido metabolism | LC/MS pos | 0.87 |
| 4-methyl-2-oxopentanoate | Amino acid | Valine, leucine and isoleucine metabolism | LC/MS neg | 25.91 |
| 5-oxoproline | Amino acid | Glutathione metabolism | LC/MS neg | 0 |
| alanine | Amino acid | Alanine and aspartate metabolism | GC/MS | 0 |
| alpha-hydroxyisocaproate | Amino acid | Valine, leucine and isoleucine metabolism | LC/MS neg | 48.84 |
| alpha-hydroxyisovalerate | Amino acid | Valine, leucine and isoleucine metabolism | LC/MS neg | 0 |
| anthranilate | Amino acid | Tryptophan metabolism | LC/MS pos | 15.94 |
| arginine | Amino acid | Urea cycle; arginine-, proline-, metabolism | LC/MS neg | 3.49 |
| asparagine | Amino acid | Alanine and aspartate metabolism | GC/MS | 16.89 |
| aspartate | Amino acid | Alanine and aspartate metabolism | LC/MS neg | 0.66 |
| beta-hydroxyisovalerate | Amino acid | Valine, leucine and isoleucine metabolism | LC/MS neg | 0.36 |
| betaine | Amino acid | Glycine, serine and threonine metabolism | LC/MS pos | 0 |
| C-glycosyltryptophan | Amino acid | Tryptophan metabolism | LC/MS pos | 0.07 |
| citrulline | Amino acid | Urea cycle; arginine-, proline-, metabolism | LC/MS pos | 2.47 |
| creatine | Amino acid | Creatine metabolism | LC/MS pos | 0 |
| creatinine | Amino acid | Creatine metabolism | LC/MS pos | 0 |
| cysteine | Amino acid | Cysteine, methionine, SAM, taurine metabolism | GC/MS | 31.22 |
| dimethylarginine (SDMA + ADMA) | Amino acid | Urea cycle; arginine-, proline-, metabolism | LC/MS pos | 0.95 |
| dimethylglycine | Amino acid | Glycine, serine and threonine metabolism | GC/MS | 19.43 |
| glutamate | Amino acid | Glutamate metabolism | LC/MS neg | 0 |
| glutarate (pentanedioate) | Amino acid | Lysine metabolism | GC/MS | 18.56 |
| glutaroyl carnitine | Amino acid | Lysine metabolism | LC/MS pos | 0.66 |
| glycine | Amino acid | Glycine, serine and threonine metabolism | GC/MS | 0 |
| histidine | Amino acid | Histidine metabolism | LC/MS neg | 0 |
| homocitrulline | Amino acid | Urea cycle; arginine-, proline-, metabolism | LC/MS pos | 14.85 |
| homostachydrine | Amino acid | Urea cycle; arginine-, proline-, metabolism | LC/MS pos | 1.02 |
| hydroxyisovaleroyl carnitine | Amino acid | Valine, leucine and isoleucine metabolism | LC/MS pos | 13.9 |
| indoleacetate | Amino acid | Tryptophan metabolism | LC/MS pos | 0.22 |
| indolelactate | Amino acid | Tryptophan metabolism | LC/MS pos | 13.61 |
| indolepropionate | Amino acid | Tryptophan metabolism | LC/MS neg | 7.13 |
| isobutyrylcarnitine | Amino acid | Valine, leucine and isoleucine metabolism | LC/MS pos | 0.22 |
| isoleucine | Amino acid | Valine, leucine and isoleucine metabolism | LC/MS pos | 0 |
| isovalerylcarnitine | Amino acid | Valine, leucine and isoleucine metabolism | LC/MS pos | 0.07 |
| kynurenine | Amino acid | Tryptophan metabolism | LC/MS pos | 0 |
| leucine | Amino acid | Valine, leucine and isoleucine metabolism | LC/MS pos | 0 |
| lysine | Amino acid | Lysine metabolism | LC/MS pos | 0 |
| methionine | Amino acid | Cysteine, methionine, SAM, taurine metabolism | LC/MS neg | 0.15 |
| methionine sulfoxide | Amino acid | Cysteine, methionine, SAM, taurine metabolism | LC/MS pos | 0.36 |
| N6-acetyllysine | Amino acid | Lysine metabolism | LC/MS pos | 0.07 |
| N-acetylalanine | Amino acid | Alanine and aspartate metabolism | LC/MS neg | 0.15 |
| N-acetyl-beta-alanine | Amino acid | Alanine and aspartate metabolism | LC/MS pos | 0.8 |
| N-acetylglycine | Amino acid | Glycine, serine and threonine metabolism | GC/MS | 26.71 |
| N-acetylornithine | Amino acid | Urea cycle; arginine-, proline-, metabolism | LC/MS pos | 1.24 |
| N-acetylphenylalanine | Amino acid | Phenylalanine & tyrosine metabolism | LC/MS neg | 6.84 |
| N-acetylserine | Amino acid | Glycine, serine and threonine metabolism | LC/MS pos | 24.6 |
| N-acetylthreonine | Amino acid | Glycine, serine and threonine metabolism | LC/MS neg | 2.47 |
| N-methyl proline | Amino acid | Urea cycle; arginine-, proline-, metabolism | LC/MS pos | 16.81 |
| o-cresol sulfate | Amino acid | Phenylalanine & tyrosine metabolism | LC/MS neg | 25.18 |
| ornithine | Amino acid | Urea cycle; arginine-, proline-, metabolism | GC/MS | 2.69 |
| p-cresol sulfate | Amino acid | Phenylalanine & tyrosine metabolism | LC/MS neg | 0 |
| phenol sulfate | Amino acid | Phenylalanine & tyrosine metabolism | LC/MS neg | 0 |
| phenylacetate | Amino acid | Phenylalanine & tyrosine metabolism | LC/MS neg | 18.41 |
| phenylacetylglutamine | Amino acid | Phenylalanine & tyrosine metabolism | LC/MS pos | 0.07 |
| phenylalanine | Amino acid | Phenylalanine & tyrosine metabolism | LC/MS pos | 0 |
| phenyllactate (PLA) | Amino acid | Phenylalanine & tyrosine metabolism | LC/MS neg | 14.92 |
| pipecolate | Amino acid | Lysine metabolism | LC/MS pos | 0.87 |
| proline | Amino acid | Urea cycle; arginine-, proline-, metabolism | LC/MS pos | 0 |
| pyroglutamine | Amino acid | Glutamate metabolism | LC/MS pos | 0.58 |
| serine | Amino acid | Glycine, serine and threonine metabolism | GC/MS | 0 |
| serotonin (5HT) | Amino acid | Tryptophan metabolism | LC/MS pos | 1.6 |
| stachydrine | Amino acid | Urea cycle; arginine-, proline-, metabolism | LC/MS pos | 0.36 |
| threonine | Amino acid | Glycine, serine and threonine metabolism | LC/MS pos | 0.29 |
| tiglyl carnitine | Amino acid | Valine, leucine and isoleucine metabolism | LC/MS pos | 28.31 |
| trans-4-hydroxyproline | Amino acid | Urea cycle; arginine-, proline-, metabolism | LC/MS pos | 0.58 |
| tryptophan | Amino acid | Tryptophan metabolism | LC/MS pos | 0 |
| tryptophan betaine | Amino acid | Tryptophan metabolism | LC/MS pos | 1.02 |
| tyrosine | Amino acid | Phenylalanine & tyrosine metabolism | LC/MS pos | 0 |
| urea | Amino acid | Urea cycle; arginine-, proline-, metabolism | GC/MS | 0 |
| urocanate | Amino acid | Histidine metabolism | LC/MS pos | 3.28 |
| valine | Amino acid | Valine, leucine and isoleucine metabolism | LC/MS pos | 0 |
| 1,5-anhydroglucitol (1,5-AG) | Carbohydrate | Glycolysis, gluconeogenesis, pyruvate metabolism | GC/MS | 2.04 |
| 1,6-anhydroglucose | Carbohydrate | Glycolysis, gluconeogenesis, pyruvate metabolism | GC/MS | 41.05 |
| arabinose | Carbohydrate | Nucleotide sugars, pentose metabolism | GC/MS | 16.59 |
| erythronate | Carbohydrate | Aminosugars metabolism | GC/MS | 1.38 |
| erythrose | Carbohydrate | Fructose, mannose, galactose, starch, and sucrose metabolism | GC/MS | 10.41 |
| fructose | Carbohydrate | Fructose, mannose, galactose, starch, and sucrose metabolism | GC/MS | 0 |
| gluconate | Carbohydrate | Nucleotide sugars, pentose metabolism | GC/MS | 19.87 |
| glucose | Carbohydrate | Glycolysis, gluconeogenesis, pyruvate metabolism | GC/MS | 0 |
| glucuronate | Carbohydrate | Glycolysis, gluconeogenesis, pyruvate metabolism | GC/MS | 34.57 |
| glycerate | Carbohydrate | Glycolysis, gluconeogenesis, pyruvate metabolism | GC/MS | 0.15 |
| lactate | Carbohydrate | Glycolysis, gluconeogenesis, pyruvate metabolism | GC/MS | 0 |
| mannitol | Carbohydrate | Fructose, mannose, galactose, starch, and sucrose metabolism | GC/MS | 38.28 |
| mannose | Carbohydrate | Fructose, mannose, galactose, starch, and sucrose metabolism | GC/MS | 0.07 |
| pyruvate | Carbohydrate | Glycolysis, gluconeogenesis, pyruvate metabolism | GC/MS | 50 |
| threitol | Carbohydrate | Nucleotide sugars, pentose metabolism | GC/MS | 25.98 |
| trehalose | Carbohydrate | Fructose, mannose, galactose, starch, and sucrose metabolism | GC/MS | 20.31 |
| alpha-tocopherol | Cofactors and vitamins | Tocopherol metabolism | GC/MS | 23.36 |
| arabonate | Cofactors and vitamins | Ascorbate and aldarate metabolism | GC/MS | 4.51 |
| bilirubin (E,E) | Cofactors and vitamins | Hemoglobin and porphyrin metabolism | LC/MS neg | 6.4 |
| bilirubin (Z,Z) | Cofactors and vitamins | Hemoglobin and porphyrin metabolism | LC/MS neg | 45.78 |
| biliverdin | Cofactors and vitamins | Hemoglobin and porphyrin metabolism | LC/MS pos | 34.72 |
| gamma-tocopherol | Cofactors and vitamins | Tocopherol metabolism | GC/MS | 32.75 |
| pantothenate | Cofactors and vitamins | Pantothenate and CoA metabolism | LC/MS pos | 1.24 |
| pyridoxate | Cofactors and vitamins | Vitamin B6 metabolism | LC/MS neg | 0.07 |
| threonate | Cofactors and vitamins | Ascorbate and aldarate metabolism | GC/MS | 4 |
| acetylphosphate | Energy | Oxidative phosphorylation | GC/MS | 0 |
| cis-aconitate | Energy | Krebs cycle | LC/MS neg | 0 |
| citrate | Energy | Krebs cycle | GC/MS | 0 |
| malate | Energy | Krebs cycle | GC/MS | 15.65 |
| phosphate | Energy | Oxidative phosphorylation | GC/MS | 0 |
| succinate | Energy | Krebs cycle | LC/MS neg | 0 |
| succinylcarnitine | Energy | Krebs cycle | LC/MS pos | 4.44 |
| 1,2 propanediol | Lipid | Ketone bodies | GC/MS | 0.51 |
| 10-heptadecenoate (17:1n7) | Lipid | Long chain fatty acid | LC/MS neg | 0 |
| 10-nonadecenoate (19:1n9) | Lipid | Long chain fatty acid | LC/MS neg | 0 |
| 13-HODE + 9-HODE | Lipid | Fatty acid, monohydroxy | LC/MS neg | 0 |
| 1-arachidonoylglycerophosphocholine | Lipid | Lysolipid | LC/MS pos | 8.95 |
| 1-arachidonoylglycero  phosphoethanolamine | Lipid | Lysolipid | LC/MS neg | 0 |
| 1-arachidonoylglycerophosphoinositol | Lipid | Lysolipid | LC/MS neg | 0 |
| 1-docosahexaenoylglycero  phosphocholine | Lipid | Lysolipid | LC/MS pos | 0 |
| 1-docosapentaenoylglycerophosphocholine | Lipid | Lysolipid | LC/MS pos | 6.4 |
| 1-eicosadienoylglycerophosphocholine | Lipid | Lysolipid | LC/MS pos | 2.62 |
| 1-eicosatrienoylglycerophosphocholine | Lipid | Lysolipid | LC/MS pos | 0 |
| 1-heptadecanoylglycerophosphocholine | Lipid | Lysolipid | LC/MS pos | 0.87 |
| 1-linoleoylglycerophosphocholine | Lipid | Lysolipid | LC/MS pos | 0.29 |
| 1-linoleoylglycerophosphoethanolamine | Lipid | Lysolipid | LC/MS neg | 0 |
| 1-myristoylglycerophosphocholine | Lipid | Lysolipid | LC/MS pos | 0 |
| 1-O-hexadecylglycerophosphocholine | Lipid | Lysolipid | LC/MS pos | 0 |
| 1-oleoylglycerol (1-monoolein) | Lipid | Monoacylglycerol | LC/MS pos | 0.07 |
| 1-oleoylglycerophosphocholine | Lipid | Lysolipid | LC/MS pos | 0 |
| 1-oleoylglycerophosphoethanolamine | Lipid | Lysolipid | LC/MS neg | 0.36 |
| 1-palmitoleoylglycerophosphocholine | Lipid | Lysolipid | LC/MS pos | 0 |
| 1-palmitoylglycerol (1-monopalmitin) | Lipid | Monoacylglycerol | GC/MS | 6.55 |
| 1-palmitoylglycerophosphocholine | Lipid | Lysolipid | LC/MS pos | 0 |
| 1-palmitoylglycerophosphoethanolamine | Lipid | Lysolipid | LC/MS neg | 0 |
| 1-palmitoylglycerophosphoinositol | Lipid | Lysolipid | LC/MS neg | 0.36 |
| 1-pentadecanoylglycerophosphocholine | Lipid | Lysolipid | LC/MS pos | 2.11 |
| 1-stearoylglycerol (1-monostearin) | Lipid | Monoacylglycerol | GC/MS | 8.52 |
| 1-stearoylglycerophosphocholine | Lipid | Lysolipid | LC/MS pos | 0 |
| 1-stearoylglycerophosphoethanolamine | Lipid | Lysolipid | LC/MS neg | 1.75 |
| 1-stearoylglycerophosphoinositol | Lipid | Lysolipid | LC/MS neg | 0 |
| 21-hydroxypregnenolone disulfate | Lipid | Sterol/Steroid | LC/MS neg | 14.85 |
| 2-arachidonoylglycerophosphocholine | Lipid | Lysolipid | LC/MS pos | 30.42 |
| 2-arachidonoylglycero  phosphoethanolamine | Lipid | Lysolipid | LC/MS neg | 9.1 |
| 2-hydroxyglutarate | Lipid | Fatty acid, dicarboxylate | GC/MS | 13.68 |
| 2-hydroxyoctanoate | Lipid | Fatty acid, monohydroxy | LC/MS neg | 44.61 |
| 2-hydroxypalmitate | Lipid | Fatty acid, monohydroxy | LC/MS neg | 0 |
| 2-hydroxystearate | Lipid | Fatty acid, monohydroxy | LC/MS neg | 0 |
| 2-linoleoylglycerophosphocholine | Lipid | Lysolipid | LC/MS pos | 4.73 |
| 2-linoleoylglycerophosphoethanolamine | Lipid | Lysolipid | LC/MS neg | 24.82 |
| 2-oleoylglycerophosphocholine | Lipid | Lysolipid | LC/MS pos | 0.15 |
| 2-oleoylglycerophosphoethanolamine | Lipid | Lysolipid | LC/MS neg | 42.72 |
| 2-palmitoylglycerophosphocholine | Lipid | Lysolipid | LC/MS pos | 0 |
| 2-palmitoylglycero  phosphoethanolamine | Lipid | Lysolipid | LC/MS neg | 10.41 |
| 2-stearoylglycerophosphocholine | Lipid | Lysolipid | LC/MS pos | 0 |
| 3-carboxy-4-methyl-5-propyl-2-furanpropanoate (CMPF) | Lipid | Fatty acid, dicarboxylate | LC/MS neg | 0.07 |
| 3-dehydrocarnitine | Lipid | Carnitine metabolism | LC/MS pos | 0 |
| 3-hydroxybutyrate (BHBA) | Lipid | Ketone bodies | GC/MS | 0.07 |
| 3-hydroxydecanoate | Lipid | Fatty acid, monohydroxy | LC/MS neg | 0.36 |
| 4-androsten-3beta,  17beta-diol disulfate 1 | Lipid | Sterol/Steroid | LC/MS neg | 0.15 |
| 4-androsten-3beta,  17beta-diol disulfate 2 | Lipid | Sterol/Steroid | LC/MS neg | 0.58 |
| 5alpha-androstan-3beta,  17beta-diol disulfate | Lipid | Sterol/Steroid | LC/MS neg | 10.33 |
| 5alpha-pregnan-3beta,  20alpha-diol disulfate | Lipid | Sterol/Steroid | LC/MS neg | 12.81 |
| 5-dodecenoate (12:1n7) | Lipid | Medium chain fatty acid | LC/MS neg | 1.16 |
| 5-HETE | Lipid | Fatty acid, monohydroxy | LC/MS neg | 0.07 |
| 7-alpha-hydroxy-3-oxo-4-cholestenoate (7-Hoca) | Lipid | Sterol/Steroid | LC/MS neg | 7.42 |
| 7-beta-hydroxycholesterol | Lipid | Sterol/Steroid | GC/MS | 4.44 |
| acetylcarnitine | Lipid | Carnitine metabolism | LC/MS pos | 1.24 |
| adipate | Lipid | Fatty acid, dicarboxylate | GC/MS | 2.98 |
| adrenate (22:4n6) | Lipid | Long chain fatty acid | LC/MS neg | 0 |
| andro steroid monosulfate 2 | Lipid | Sterol/Steroid | LC/MS neg | 36.83 |
| androsterone sulfate | Lipid | Sterol/Steroid | LC/MS neg | 6.11 |
| arachidonate (20:4n6) | Lipid | Long chain fatty acid | LC/MS neg | 0 |
| azelate (nonanedioate) | Lipid | Fatty acid, dicarboxylate | LC/MS neg | 0 |
| caprate (10:0) | Lipid | Medium chain fatty acid | LC/MS neg | 0.07 |
| caproate (6:0) | Lipid | Medium chain fatty acid | LC/MS neg | 0 |
| caprylate (8:0) | Lipid | Medium chain fatty acid | LC/MS neg | 0 |
| carnitine | Lipid | Carnitine metabolism | LC/MS pos | 0 |
| cholate | Lipid | Bile acid metabolism | LC/MS neg | 42.5 |
| cholesterol | Lipid | Sterol/Steroid | GC/MS | 0 |
| choline | Lipid | Glycerolipid metabolism | LC/MS pos | 0 |
| cis-vaccenate (18:1n7) | Lipid | Long chain fatty acid | GC/MS | 2.91 |
| cortisol | Lipid | Sterol/Steroid | LC/MS pos | 0.29 |
| cortisone | Lipid | Sterol/Steroid | LC/MS pos | 1.46 |
| decanoylcarnitine | Lipid | Carnitine metabolism | LC/MS pos | 0 |
| dehydroisoandrosterone sulfate  (DHEA-S) | Lipid | Sterol/Steroid | LC/MS neg | 0.44 |
| deoxycarnitine | Lipid | Carnitine metabolism | LC/MS pos | 0 |
| deoxycholate | Lipid | Bile acid metabolism | LC/MS neg | 23.07 |
| dihomo-linoleate (20:2n6) | Lipid | Long chain fatty acid | LC/MS neg | 0 |
| dihomo-linolenate (20:3n3 or n6) | Lipid | Essential fatty acid | LC/MS neg | 0 |
| docosahexaenoate (DHA; 22:6n3) | Lipid | Essential fatty acid | LC/MS neg | 0 |
| docosapentaenoate (n3 DPA; 22:5n3) | Lipid | Essential fatty acid | LC/MS neg | 0 |
| docosapentaenoate (n6 DPA; 22:5n6) | Lipid | Essential fatty acid | LC/MS neg | 2.98 |
| dodecanedioate | Lipid | Fatty acid, dicarboxylate | LC/MS neg | 1.89 |
| eicosapentaenoate (EPA; 20:5n3) | Lipid | Essential fatty acid | LC/MS neg | 0 |
| eicosenoate (20:1n9 or 11) | Lipid | Long chain fatty acid | LC/MS neg | 0 |
| epiandrosterone sulfate | Lipid | Sterol/Steroid | LC/MS neg | 2.98 |
| glycerol | Lipid | Glycerolipid metabolism | GC/MS | 0 |
| glycerol 3-phosphate (G3P) | Lipid | Glycerolipid metabolism | GC/MS | 0.15 |
| glycerophosphorylcholine (GPC) | Lipid | Glycerolipid metabolism | LC/MS pos | 0 |
| glycochenodeoxycholate | Lipid | Bile acid metabolism | LC/MS neg | 3.78 |
| glycocholate | Lipid | Bile acid metabolism | LC/MS neg | 12.66 |
| glycocholenate sulfate | Lipid | Bile acid metabolism | LC/MS neg | 0.15 |
| glycodeoxycholate | Lipid | Bile acid metabolism | LC/MS neg | 17.47 |
| glycolithocholate sulfate | Lipid | Bile acid metabolism | LC/MS neg | 2.18 |
| heptanoate (7:0) | Lipid | Medium chain fatty acid | LC/MS neg | 0 |
| hexadecanedioate | Lipid | Fatty acid, dicarboxylate | LC/MS neg | 1.67 |
| hexanoylcarnitine | Lipid | Carnitine metabolism | LC/MS pos | 2.69 |
| hyodeoxycholate | Lipid | Bile acid metabolism | LC/MS neg | 32.75 |
| inositol 1-phosphate (I1P) | Lipid | Inositol metabolism | GC/MS | 19.94 |
| isovalerate | Lipid | Fatty acid metabolism | LC/MS neg | 0 |
| laurate (12:0) | Lipid | Medium chain fatty acid | LC/MS neg | 0 |
| laurylcarnitine | Lipid | Carnitine metabolism | LC/MS pos | 21.83 |
| linoleate (18:2n6) | Lipid | Long chain fatty acid | LC/MS neg | 0 |
| linolenate  [alpha or gamma; (18:3n3 or 6)] | Lipid | Essential fatty acid | LC/MS neg | 0 |
| margarate (17:0) | Lipid | Long chain fatty acid | LC/MS neg | 0.44 |
| methyl palmitate | Lipid | Fatty acid, methyl ester | GC/MS | 17.47 |
| myo-inositol | Lipid | Inositol metabolism | GC/MS | 0 |
| myristate (14:0) | Lipid | Long chain fatty acid | LC/MS neg | 0 |
| myristoleate (14:1n5) | Lipid | Long chain fatty acid | LC/MS neg | 0 |
| nonadecanoate (19:0) | Lipid | Long chain fatty acid | LC/MS neg | 0.07 |
| octadecanedioate | Lipid | Fatty acid, dicarboxylate | LC/MS neg | 3.35 |
| octanoylcarnitine | Lipid | Carnitine metabolism | LC/MS pos | 0 |
| oleate (18:1n9) | Lipid | Long chain fatty acid | GC/MS | 0 |
| oleoylcarnitine | Lipid | Carnitine metabolism | LC/MS pos | 0.29 |
| palmitate (16:0) | Lipid | Long chain fatty acid | LC/MS neg | 0 |
| palmitoleate (16:1n7) | Lipid | Long chain fatty acid | LC/MS neg | 0 |
| palmitoyl sphingomyelin | Lipid | Sphingolipid | GC/MS | 0.36 |
| palmitoylcarnitine | Lipid | Carnitine metabolism | LC/MS pos | 8.08 |
| pelargonate (9:0) | Lipid | Medium chain fatty acid | LC/MS neg | 0 |
| pregn steroid monosulfate | Lipid | Sterol/Steroid | LC/MS neg | 0.15 |
| pregnen-diol disulfate | Lipid | Sterol/Steroid | LC/MS neg | 0.07 |
| propionylcarnitine | Lipid | Fatty acid metabolism (also BCAA metabolism) | LC/MS pos | 0.07 |
| scyllo-inositol | Lipid | Inositol metabolism | GC/MS | 26.93 |
| sebacate (decanedioate) | Lipid | Fatty acid, dicarboxylate | LC/MS neg | 3.86 |
| stearate (18:0) | Lipid | Long chain fatty acid | LC/MS neg | 0 |
| stearidonate (18:4n3) | Lipid | Long chain fatty acid | LC/MS neg | 1.16 |
| stearoyl sphingomyelin | Lipid | Sphingolipid | GC/MS | 6.04 |
| stearoylcarnitine | Lipid | Carnitine metabolism | LC/MS pos | 25.91 |
| suberate (octanedioate) | Lipid | Fatty acid, dicarboxylate | LC/MS neg | 3.35 |
| taurochenodeoxycholate | Lipid | Bile acid metabolism | LC/MS neg | 28.75 |
| taurocholate | Lipid | Bile acid metabolism | LC/MS neg | 48.47 |
| taurocholenate sulfate | Lipid | Bile acid metabolism | LC/MS neg | 7.13 |
| taurolithocholate 3-sulfate | Lipid | Bile acid metabolism | LC/MS neg | 16.01 |
| tetradecanedioate | Lipid | Fatty acid, dicarboxylate | LC/MS neg | 19.14 |
| undecanedioate | Lipid | Fatty acid, dicarboxylate | LC/MS neg | 3.49 |
| undecanoate (11:0) | Lipid | Medium chain fatty acid | LC/MS neg | 0.15 |
| valerate | Lipid | Short chain fatty acid | LC/MS neg | 2.33 |
| 5-methyluridine (ribothymidine) | Nucleotide | Pyrimidine metabolism, uracil containing | LC/MS neg | 13.03 |
| 7-methylguanine | Nucleotide | Purine metabolism, guanine containing | LC/MS pos | 5.75 |
| adenosine | Nucleotide | Purine metabolism, adenine containing | LC/MS pos | 33.41 |
| allantoin | Nucleotide | Purine metabolism, urate metabolism | GC/MS | 38.57 |
| guanosine | Nucleotide | Purine metabolism, guanine containing | LC/MS pos | 5.31 |
| hypoxanthine | Nucleotide | Purine metabolism, (hypo)xanthine/inosine containing | LC/MS neg | 0.73 |
| inosine | Nucleotide | Purine metabolism, (hypo)xanthine/inosine containing | LC/MS neg | 2.18 |
| N1-methyladenosine | Nucleotide | Purine metabolism, adenine containing | LC/MS pos | 3.86 |
| pseudouridine | Nucleotide | Pyrimidine metabolism, uracil containing | LC/MS pos | 0 |
| urate | Nucleotide | Purine metabolism, urate metabolism | LC/MS neg | 0 |
| uridine | Nucleotide | Pyrimidine metabolism, uracil containing | LC/MS neg | 0 |
| xanthine | Nucleotide | Purine metabolism, (hypo)xanthine/inosine containing | LC/MS pos | 0.07 |
| [H]HWESASLLR[OH] | Peptide | Polypeptide | LC/MS pos | 7.42 |
| alanylleucine | Peptide | Dipeptide | LC/MS pos | 22.71 |
| alpha-glutamylglutamate | Peptide | Dipeptide | LC/MS pos | 41.48 |
| aspartylphenylalanine | Peptide | Dipeptide | LC/MS pos | 0 |
| bradykinin, des-arg(9) | Peptide | Polypeptide | LC/MS pos | 9.32 |
| DSGEGDFXAEGGGVR | Peptide | Fibrinogen cleavage peptide | LC/MS pos | 3.06 |
| gamma-glutamylalanine | Peptide | gamma-glutamyl | LC/MS pos | 4.37 |
| gamma-glutamylglutamate | Peptide | gamma-glutamyl | LC/MS pos | 1.02 |
| gamma-glutamylisoleucine | Peptide | gamma-glutamyl | LC/MS pos | 0 |
| gamma-glutamylleucine | Peptide | gamma-glutamyl | LC/MS pos | 0 |
| gamma-glutamylphenylalanine | Peptide | gamma-glutamyl | LC/MS pos | 0 |
| gamma-glutamylthreonine | Peptide | gamma-glutamyl | LC/MS pos | 0.22 |
| gamma-glutamyltyrosine | Peptide | gamma-glutamyl | LC/MS pos | 0.15 |
| gamma-glutamylvaline | Peptide | gamma-glutamyl | LC/MS pos | 0 |
| glycylleucine | Peptide | Dipeptide | LC/MS pos | 1.16 |
| glycylphenylalanine | Peptide | Dipeptide | LC/MS neg | 7.13 |
| glycyltyrosine | Peptide | Dipeptide | LC/MS pos | 27.95 |
| glycylvaline | Peptide | Dipeptide | LC/MS pos | 0 |
| HWESASXX | Peptide | Polypeptide | LC/MS pos | 0 |
| HXGXA | Peptide | Polypeptide | LC/MS pos | 1.75 |
| leucylleucine | Peptide | Dipeptide | LC/MS pos | 0.07 |
| leucylphenylalanine | Peptide | Dipeptide | LC/MS neg | 0.22 |
| pro-hydroxy-pro | Peptide | Dipeptide | LC/MS pos | 0.22 |
| pyroglutamylglycine | Peptide | Dipeptide | LC/MS neg | 11.06 |
| threonylphenylalanine | Peptide | Dipeptide | LC/MS pos | 0.66 |
| 1,7-dimethylurate | Xenobiotics | Xanthine metabolism | LC/MS neg | 38.72 |
| 1-methylurate | Xenobiotics | Xanthine metabolism | LC/MS neg | 31.59 |
| 2-hydroxyhippurate (salicylurate) | Xenobiotics | Benzoate metabolism | LC/MS neg | 35.88 |
| 3-ethylphenylsulfate | Xenobiotics | Benzoate metabolism | LC/MS neg | 24.53 |
| 4-ethylphenylsulfate | Xenobiotics | Benzoate metabolism | LC/MS neg | 44.98 |
| 4-hydroxyhippurate | Xenobiotics | Benzoate metabolism | LC/MS neg | 17.54 |
| 4-vinylphenol sulfate | Xenobiotics | Benzoate metabolism | LC/MS neg | 4.51 |
| 5-acetylamino-6-amino-3-methyluracil | Xenobiotics | Xanthine metabolism | LC/MS neg | 39.67 |
| benzoate | Xenobiotics | Benzoate metabolism | GC/MS | 0 |
| caffeine | Xenobiotics | Xanthine metabolism | LC/MS pos | 11.43 |
| catechol sulfate | Xenobiotics | Benzoate metabolism | LC/MS neg | 0 |
| erythritol | Xenobiotics | Sugar, sugar substitute, starch | GC/MS | 0.51 |
| glycerol 2-phosphate | Xenobiotics | Chemical | GC/MS | 23.14 |
| hippurate | Xenobiotics | Benzoate metabolism | LC/MS neg | 0 |
| paraxanthine | Xenobiotics | Xanthine metabolism | LC/MS pos | 9.17 |
| piperine | Xenobiotics | Food component/Plant | LC/MS pos | 0.87 |
| salicylate | Xenobiotics | Drug | LC/MS neg | 12.88 |
| theobromine | Xenobiotics | Xanthine metabolism | LC/MS pos | 11.64 |
| theophylline | Xenobiotics | Xanthine metabolism | LC/MS neg | 22.93 |
| thymol sulfate | Xenobiotics | Food component/Plant | LC/MS neg | 27.44 |
