# Supplementary material for: Genetic Determinants Influencing Human Serum Metabolome among African Americans
Source: PLoS Genet. 2014 Mar 13;10(3):e1004212. doi: 10.1371/journal.pgen.1004212 (PMC3952826; doi:10.1371/journal.pgen.1004212)
Supplement: Table S2 — Baseline characteristics of African-Americans in ARIC for genetic analyses. (DOCX) [file pgen.1004212.s005.docx]

**Table S2**. Baseline Characteristics of African Americans in ARIC for these genome-wide association analyses

|  | **Genome-wide association study**  **(N = 1,260)** |
| --- | --- |
| Age (y) | 52.6 ± 5.6 |
| Male (%) | 34.8 |
| BMI (kg/m^2^) | 29.7 ± 6.0 |
| Hypertension (%) | 54.1 |
| Diabetes (%) | 15.1 |
| Prevalent CHD (%) | 3.7 |
| Current smoking (%) | 28.5 |
| SBP (mm Hg) | 127.7 ± 20.9 |
| DBP (mm Hg) | 80.6 ± 12.0 |
| HDL cholesterol (mg/dL) | 55.5 ± 16.9 |
| LDL cholesterol (mg/dL) | 138.8 ± 42.9 |
| Triglycerides (mg/dL) | 108.5 ± 59.2 |
| Total cholesterol (mg/dL) | 215.8 ± 44.6 |
| eGFR, mL/min/1.73 m^2^ | 104.9 ± 17.1 |

BMI indicates body mass index; CHD, coronary heart disease; SBP, systolic blood pressure; DBP, diastolic blood pressure; HDL, high-density lipoprotein; LDL, low-density lipoprotein and eGFR, estimated glomerular filtration rate.

For continuous variables, mean values ± standard errors are shown. Categorical variables are given as percentage.
