# Supplementary material for: Genetic Determinants Influencing Human Serum Metabolome among African Americans
Source: PLoS Genet. 2014 Mar 13;10(3):e1004212. doi: 10.1371/journal.pgen.1004212 (PMC3952826; doi:10.1371/journal.pgen.1004212)
Supplement: Table S3 — A comparison of common variant-metabolite association among ARIC, KORA and TwinsUK studies. (DOCX) [file pgen.1004212.s006.docx]

**Table S3**. A comparison of common variant-metabolite association among ARIC, KORA and TwinsUK studies

| **Metabolites** | **ARIC** | | **KORA** | | **TwinsUK** | |
| --- | --- | --- | --- | --- | --- | --- |
|  | **Top SNP** | **P** | **Top SNP** | **P** | **Top SNP** | **P** |
| creatine | rs2433610  15kb from *GATM* | 9🞨10^-12^ | rs3111102  not in LD | 5🞨10^-6^ | rs1860867  not in LD | 2🞨10^-6^ |
| phenylacetate | rs7499271  *ACSM2B* (intron) | 6🞨10^-11^ | rs358749  not in LD | 2🞨10^-7^ | rs4243233 not in LD | 8🞨10^-8^ |
| acetylcarnitine | rs12282107  *SIAE* (missense) | 8🞨10^-14^ | rs1632879  not in LD | 7🞨10^-7^ | rs160851  not in LD | 1🞨10^-6^ |
| hexadecanedioate | rs17028615  6 kb from *ADH4* | 2🞨10^-15^ | rs1282  not in LD | 3🞨10^-6^ | rs7571804 not in LD | 6🞨10^-6^ |
