# Supplementary material for: Genetic Determinants Influencing Human Serum Metabolome among African Americans
Source: PLoS Genet. 2014 Mar 13;10(3):e1004212. doi: 10.1371/journal.pgen.1004212 (PMC3952826; doi:10.1371/journal.pgen.1004212)
Supplement: Table S4 — Association between genome-wide significant metabolites and clinical endpoints among African-Americans in ARIC. (DOCX) [file pgen.1004212.s007.docx]

**Table S4**. Association between genome-wide significant metabolites and clinical endpoints among ARIC African-Americans

| Metabolite | Risk Factor | Clinical Endpoint | N | Beta | HR | SE | P | Design |
| --- | --- | --- | --- | --- | --- | --- | --- | --- |
| [H]HWESASLLR[OH] | DBP | / | 919 | 0.75 | / | 0.29 | 0.008 | cross-sectional |
| Threonylphenylalanine | DBP | / | 919 | -1.44 | / | 0.44 | 0.001 | cross-sectional |
| Glycine | fibrinogen | / | 1823 | -11.71 | / | 5.40 | 0.03 | cross-sectional |
| N-acetylornithine | eGFR  _CKD-EPI_ | / | 1921 | -5.08 | / | 0.71 | 9.0×10^-13^ | cross-sectional |
| N-acetylornithine | / | incident CKD | 1921 | 0.49 | 1.64 | 0.17 | 0.003 | longitudinal |
| N-acetyl-1-methylhistidine | eGFR  _CKD-EPI_ | / | 1921 | -5.72 | / | 0.59 | 1.6×10^-21^ | cross-sectional |
| N-acetyl-1-methylhistidine | / | incident CKD | 1921 | 0.29 | 1.34 | 0.13 | 0.03 | longitudinal |
| trehalose | glucose | / | 1656 | 1.97 | / | 0.23 | 2.9×10^-17^ | cross-sectional |
| trehalose | / | incident T2D | 1430 | 0.29 | 1.34 | 0.07 | 2.0×10^-5^ | longitudinal |

N indicates sample size; SD, standard deviation; HR, hazard ratio; SE standard error; DBP, diastolic blood pressure; eGFR_CKD-EPI_, estimate glomerular filtration rate calculated by The Chronic Kidney Disease Epidemiology Collaboration equation; SBP, systolic blood pressure, HDL, high-density lipoprotein; LDL, low-density lipoprotein; CHD, coronary heart disease; CKD, chronic kidney disease; T2D, type 2 diabetes; BMI, body mass index. All metabolites values were natural log-transformed prior to the analyses.

Metabolite levels were treated continuously and were centered by its mean and scaled by its standard deviation. Linear regressions were applied to estimate the cross-sectional association and Cox proportional hazard regressions were conducted to estimate the longitudinal association. For the longitudinal analysis of incident disease endpoints, prevalent cases were excluded. In addition, prevalent hypertension cases were excluded for the diastolic blood pressure analyses; prevalent coronary heart disease (CHD) cases were excluded for the fibrinogen analysis; prevalent chronic kidney disease (CKD) cases were excluded for the estimated Glomerular Filtration Rate (eGFR) analysis; and prevalent type 2 diabetes cases were excluded for the glucose analysis.

Incident CKD was defined previously in those with a baseline eGFRCKD-EPI ≥ 60mL/min/1.73m2 as either: at least one follow-up eGFRCKD-EPI < 60 mL/min/1.73m2 and < 75% of baseline (i.e., >=25% decrease); or the individual had at least one surveillance-based CKD event (hospitalization or CKD-related death) after baseline up to 2008. Incident diabetes was defined in those free of diabetes at baseline and having a follow-up visit with either: (1) a fasting glucose ≥7.0 mmol/L, (2) a non-fasting glucose ≥11.1 mmol/L, (3) use of a diabetes medication, or (4) self-reported physician diagnosis.
