# Supplementary material for: Genetic Determinants Influencing Human Serum Metabolome among African Americans
Source: PLoS Genet. 2014 Mar 13;10(3):e1004212. doi: 10.1371/journal.pgen.1004212 (PMC3952826; doi:10.1371/journal.pgen.1004212)
Supplement: Table S5 — Baseline characteristics of African-Americans in ARIC for incident disease association analyses. (DOCX) [file pgen.1004212.s008.docx]

**Table S5**. Baseline Characteristics of African-Americans in ARIC for incident disease association analyses

|  | **Incident CKD analysis**  **(N = 1,921)** | |  | **Incident T2D analysis**  **(N=1,430)** | |
| --- | --- | --- | --- | --- | --- |
|  | CKD cases | Non-CKD cases |  | T2D cases | Non-T2D cases |
| N | 204 | 1717 |  | 255 | 1175 |
| Age (y) | 54.4 ± 5.6 | 52.6 ± 5.7 |  | 52.8 ± 5.5 | 52.2 ± 5.7 |
| Male (%) | 42.2 | 35.0 |  | 34.1 | 36.7 |
| BMI (kg/m^2^) | 30.6 ± 6.0 | 29.5 ± 6.0 |  | 31.6 ± 6.4 | 28.7 ± 5.8 |
| Hypertension (%) | 65.7 | 51.2 |  | 60.0 | 47.8 |
| Diabetes (%) | 37.7 | 13.0 |  | / | / |
| Prevalent CHD (%) | 6.4 | 3.3 |  | 2.0 | 3.0 |
| Current smoking (%) | 32.4 | 28.4 |  | 26.4 | 28.8 |
| SBP (mm Hg) | 133.6 ± 22.3 | 127.2 ± 20.8 |  | 128.8 ± 19.5 | 126.0 ± 19.9 |
| DBP (mm Hg) | 81.6 ± 10.2 | 80.1 ± 12.0 |  | 81.1 ± 11.5 | 80.2 ± 11.7 |
| HDL cholesterol (mg/dL) | 51.5 ± 17.0 | 56.0 ± 17.2 |  | 52.1 ± 15.3 | 58.0 ± 17.9 |
| LDL cholesterol (mg/dL) | 144.2 ± 47.8 | 137.4 ± 42.5 |  | 136.6 ± 40.6 | 136.7 ± 43.8 |
| Triglycerides (mg/dL) | 127.7 ± 68.8 | 105.0 ± 55.6 |  | 116.0 ± 53.7 | 97.7 ± 47.7 |
| Total cholesterol (mg/dL) | 220.5 ± 51.5 | 214.3 ± 44.1 |  | 211.9 ± 41.9 | 214.3 ± 44.9 |
| eGFR, mL/min/1.73 m^2^ | 97.5 ± 17.6 | 106.2 ± 15.9 |  | 104.8 ± 16.4 | 104.7 ± 17.2 |

CKD indicates chronic kidney disease; T2D, type 2 diabetes; BMI, body mass index; CHD, coronary heart disease; SBP, systolic blood pressure; DBP, diastolic blood pressure; HDL, high-density lipoprotein; LDL, low-density lipoprotein and eGFR, estimated glomerular filtration rate.

For continuous variables, mean values ± standard errors are shown. Categorical variables are given as percentage.
